# The effects of eating frequency on changes in body composition and cardiometabolic health in adults: a systematic review with meta-analysis of randomized trials

**DOI:** 10.1186/s12966-023-01532-z

**Published:** 2023-11-14

**Authors:** Paul Blazey, Alireza Habibi, Nejat Hassen, Daniel Friedman, Karim M. Khan, Clare L. Ardern

**Affiliations:** 1 Department of Family Practice, Faculty of Medicine, University of British Columbia, Vancouver, Canada; 2 Centre for Aging SMART at Vancouver Coastal Health, Vancouver Coastal Health Research Institute, Vancouver, Canada; 3 Faculty of Medicine, University of British Columbia, Vancouver, Canada; 4 Arthritis Research Canada, Vancouver, BC Canada; 5 AIS Medicine, Australian Institute of Sport, Canberra, Australian Capital Territory Australia; 6 School of Kinesiology, The University of British Columbia, Vancouver, BC Canada; 7 Sport and Exercise Medicine Research Centre, La Trobe University, Melbourne, Australia; 8 Department of Physical Therapy, University of British Columbia, Vancouver, Canada

**Keywords:** Eating frequency, Feeding behavior, Diet, Cardiometabolic health, Weight change, Dietary pattern

## Abstract

**Background:**

Eating frequency may affect body weight and cardiometabolic health. Intervention trials and observational studies have both indicated that high- and low-frequency eating can be associated with better health outcomes. There are currently no guidelines to inform how to advise healthy adults about how frequently to consume food or beverages.

**Aim:**

To establish whether restricted- (≤ three meals per day) frequency had a superior effect on markers of cardiometabolic health (primary outcome: weight change) compared to unrestricted-eating (≥ four meals per day) frequency in adults.

**Methods:**

We searched Medline (Ovid), Embase, CINAHL (EBSCO), Cochrane Central Register of Controlled Trials (CENTRAL), CAB Direct and Web of Science Core Collection electronic databases from inception to 7 June 2022 for clinical trials (randomised parallel or cross-over trials) reporting on the effect of high or low-frequency eating on cardiometabolic health (primary outcome: weight change). Trial interventions had to last for at least two weeks, and had to have been conducted in human adults. Bias was assessed using the Cochrane Risk of Bias tool 2.0. Standardized mean differences (SMD) and 95% confidence intervals were calculated for all outcomes. Certainty of the evidence was assessed using the Grading of Recommendations Assessment, Development, and Evaluation (GRADE) approach.

**Results:**

Seventeen reports covering 16 trials were included in the systematic review. Data from five trials were excluded from meta-analysis due to insufficient reporting. 15 of 16 trials were at high risk of bias. There was very low certainty evidence of no difference between high- and low-frequency eating for weight-change (MD: -0.62 kg, CI^95^: -2.76 to 1.52 kg, *p* = 0.57).

**Conclusions:**

There was no discernible advantage to eating in a high- or low-frequency dietary pattern for cardiometabolic health. We cannot advocate for either restricted- or unrestricted eating frequency to change markers of cardiometabolic health in healthy young to middle-aged adults.

**Protocol registration:**

CRD42019137938.

**Supplementary Information:**

The online version contains supplementary material available at 10.1186/s12966-023-01532-z.

## Introduction

Eating frequency (which includes drinking) can impact on a person’s health, but the public still lack clear guidance on whether eating more or less often is beneficial for health [1–3]. Eating frequency, and dietary patterns in general, are grounded in long-held societal norms, which have habituated people in the western world to a pattern of three daily meals (breakfast, lunch and dinner) plus or minus snacks [4, 5]. The three meal pattern is not necessarily based upon hunger or optimal health, but is likely to be driven by prevailing environmental and sociocultural norms [6]. Fasting—foregoing food or calorie containing drinks for long periods—has a growing body of evidence supporting health-promoting benefits, but fasting may be difficult to implement [5, 7–11].

Studies on the effect of eating frequency date back as far as 1964 [12, 13] and the literature is conflicting about whether eating very often or sporadically improves bodyweight and other markers of cardiometabolic health. Eating energy dense foods more frequently—often linked to snacking—has been associated with a propensity to overconsume calories [6, 14–18]; higher eating frequency may increase body mass (gaining more than 5 kg over a 10 year period) [19]. Observational studies, including one of over 50,000 adults, suggest that a low meal frequency reduces body mass index (BMI) [20].

In contrast, a systematic review of participants who ate between one and 24 meals per day, found no association between eating frequency and bodyweight [21]. Paradoxically, higher eating frequency reduced the risk of obesity [22], and was associated with an overall healthy lifestyle in middle-aged men and women. In a meta-analysis designed to assess the effect of eating frequency on lean body mass, high eating frequency reduced body mass, and protected lean body mass. This suggests that frequent eating may confer specific benefits in certain populations (e.g. athletes) [23].

For the public, knowing whether to eat only during traditional meal times (three times per day), or whether to spread calorie intake more frequently over the day could support simple, and practicable dietary guidance. Therefore, we conducted a systematic review of adult human intervention studies to determine whether there are cardiometabolic benefits (listed below) to either a low (three or fewer meals per day) or high (four or more meals per day) eating frequency.

## Methods

Our systematic review was reported according to the Preferred Reporting Items for Systematic Review and Meta-Analysis—PRISMA [24] guidelines and was prospectively registered on PROSPERO (CRD42019137938) [24].

### Definitions

The American Heart Association attempted to define an eating episode in their scientific statement of 2017 by suggesting that all eating occasions with any food or drink amounting to more than 210 kJ (or 50 kcals) and with no longer than 15 min of time elapsed be defined as a single episode [3]. However, due to the heterogeneity and lack of definitions in previous research we made a pragmatic decision to define an eating occasion as any ingestion of kcals, consumed either by food or beverage, with each single episode lasting no longer than one hour (including all snacks, drinks and other instances of calorific intake).

Low (three meals or less) and high (four meals or more) meal frequencies were classified to assess whether differences in health exist based upon a current conventional (three meals per day) Western-diet. Although there is overlap between fasting and low eating frequency, we considered fasting as going more than 24 h without food or drink. We considered full-day fasting to be a distinct more extreme intervention that simply reducing the frequency of meals, trials that limited intake to less than one meal per day were therefore not included in our review.

### Search strategy

We searched the Medline (Ovid), Embase, CINAHL (EBSCO), Cochrane Central Register of Controlled Trials (CENTRAL), CAB Direct and Web of Science Core Collection electronic databases from inception to 7 June 2022. Searches were also conducted of ProQuest Dissertations & Theses Global and ClinicialTrials.gov registry for grey literature for the same date range. Google Scholar was searched on 7 June 2022: we screened the first 10 pages of search records returned for relevant literature [25]. The search strategy for all databases, including keywords and MeSH terms, as well as the grey literature searches can be found in Supplementary file 1. Methodological and population search filters were used to retrieve only randomized controlled trials and to exclude pediatric and animal studies. The search filters can be found in Supplementary file 2. We supplemented the bibliographic database search with backward citation tracking.

### Trial selection

The selection criteria are listed in Table 1. Where multiple reports related to a single trial were published, both reports were included if they reported different sets of data that met our inclusion criteria. If two records reported the same data, we included data from the report that was published first in-print.

**Table 1:** Inclusion and exclusion criteria

**Inclusion criteria**
General populations of free-living adults (of ≥ 18 years of age)
Clinical trial comparing interventions of low (three or less) vs high (four or more) meal frequency (Randomized controlled trial, crossover or parallel controlled trial)
Reported an outcome related to body composition (e.g. bodyweight, BMI) and/or a surrogate marker of cardio-metabolic disease (e.g. fasting blood glucose, Hemoglobin A1c (HbA1c), lipids)
**Exclusion criteria**
Studies of participants with altered body composition due to external factors, including, but not limited to pregnancy, malignancy, eating disorders, smokers, elite athletes, or a history of bariatric surgery
Interventions of less than two weeks duration
Trials focussed on fasting for longer than 24 h (explicitly less than one meal per day)
Cross-over studies with a washout period of less than one week
Uncontrolled medication use between intervention groups (e.g. one trial arm using medications known to effect weight change)
Undefined eating frequency observed by participants
Measurements of body composition were not standardised
Inclusion of dietary supplements, pharmacological interventions or commercial diet replacement foods as part of the main intervention
Conference abstracts or proceedings papers

If studies were not available in English, they were translated using Google Translate. We used Covidence (Covidence systematic review software, Veritas Health Innovation, Melbourne, Australia; available at www.covidence.org) to manage the screening and selection process. Articles were de-duplicated, then screened.

Two reviewers independently screened titles and abstracts for inclusion; disagreements were resolved by consensus or a third reviewer if they could not reach consensus. Records that were obtained in full-text were also screened by two independent reviewers using the same process to resolve any disagreements [26].

### Data extraction

Data were extracted in duplicate and independently by two reviewers using a custom data extraction sheet. Any disagreements were resolved via consensus. We extracted the following summary data from each record:

**Table:** 

Year of publication
First author name
Manuscript title
Trial design (crossover or parallel)
Trial duration
Sample size
Geographical and age demographics of included participants
Reported genders
Participant weight-status (e.g. under/over or normal weight) at trial initiation – Determined via BMI
Trial conditions (laboratory vs free-living)
Number of meals per day in each intervention-arm
Interventions as eucalorific or equicalorific
Diets providing energy surplus, deficit, balance or unclear
Methods to ensure dietary compliance
Methods for measuring bodyweight and mass (e.g. fat mass)
Sources of funding

### Outcome data

Primary outcome data were collected for bodyweight (kgs). Secondary outcome data were collected for:

- BMI (kg/m^2^);
- fat-mass (kgs);
- HbA1c;
- triglycerides (mmol/L);
- total cholesterol (mmol/L);
- LDL cholesterol (mmol/L);
- HDL cholesterol (mmol/L);
- glucose (mmol/L);
- and insulin (mU mL-1).

### Data management

Data for all primary and secondary outcomes were converted to standard units (e.g. kgs and mmol/l) using an online calculator (https://www.omnicalculator.com/).

Where data were unclear or missing, we contacted the corresponding author via email. Trials that reported secondary outcomes of interest in their manuscript were highlighted and their available trial protocols were checked to see if additional outcomes of interest had been collected. If appropriate, authors were then contacted to see if additional (non-reported) data were available regarding our primary or secondary outcomes of interest. If no reply was received, we allowed one week before contacting authors again. If data were missing (e.g. variance measures), then authors were also contacted for the additional statistical data. If no response was received, we considered the data were not available.

Trials that did not compare between restricted and unrestricted frequencies but otherwise met our inclusion criteria were included in the qualitative analysis but not the meta-analysis. If a trial reported at least one outcome of interest with the associated mean and standard deviation data pre- and post-intervention, then they were included in the meta-analysis. Trials that did not report pre- and post-intervention data on either of our primary or secondary outcomes were included in the qualitative data synthesis but excluded from the meta-analysis.

### Risk of bias assessment

Two reviewers independently assessed the risk of bias for each trial using the Cochrane Risk of Bias tool for randomized trials (RoB Version 2.0) [27]. We assessed bias at the trial- and domain-level. Any disagreements were resolved via consensus, or a third reviewer if consensus could not be reached.

### Statistical analysis

We classified interventions as restricted eating (three meals or less per day) or unrestricted eating (four meals or more per day). Differences in the effect of each intervention were assessed using mean differences with 95% confidence intervals where trials used the same outcome measure. Where different outcome measures (e.g. fat mass measured via Dual-Energy X-ray Absorptiometry (DEXA) scan or Bioelectrical impedance analysis (BIA)) were used, we estimated the standardized mean difference (SMD) (effect size). Where pre- and post-trial change values (mean and standard deviation) were not presented and individual trial-level data were not available, we calculated the standard error of the change using each intervention group’s reported pre- and post-trial standard deviation.

We planned a sensitivity analysis for the primary outcome data (restricting the analysis to data from parallel-groups trials) to evaluate whether trials design distorted the effect estimate. Subgroup analyses were planned to assess for differences between: parallel and crossover trials; female and male participants; normal weight (BMI 18.5–25.0) vs overweight (BMI 25.0–30.0) or obese (BMI > 30.0) participants as defined by their BMI; and whether a kcal deficit, balanced or surplus diet was prescribed in the eating frequency intervention.

All meta-analyses were carried out using RevMan V.5.3 (Copenhagen: The Nordic Cochrane Centre, The Cochrane Collaboration, 2014). We used a random-effects model for continuous outcomes and reported effect estimates with 95% confidence intervals. We selected random-effects analysis due to the expected heterogeneity in trial populations, and known heterogeneity of the prescribed interventions (included macro and micronutrient intakes). A funnel plot analysis was planned to assess publication bias across all outcomes. We used the I^2^ value to assess statistical heterogeneity, and considered < 40% as low heterogeneity; 30–60% as moderate; 50–90% as substantial; and 75–100% as considerable heterogeneity [28].

### Certainty of evidence

Two reviewers independently used the Grading of Recommendations Assessment, Development and Evaluation (GRADE) framework to judge the certainty of evidence for all outcomes included in the meta-analysis [29]. We considered risk of bias, inconsistency, imprecision, indirectness and risk of publication bias, and rated certainty of the evidence as high, moderate, low or very low for each outcome. A summary of findings Table was created using GRADEpro GDT (GRADEpro Guideline Development Tool [Software]. McMaster University and Evidence Prime, 2022. Available from gradepro.org.) to provide evidence summaries with absolute effects for each outcome [29].

## Results

**Fig. 1:**
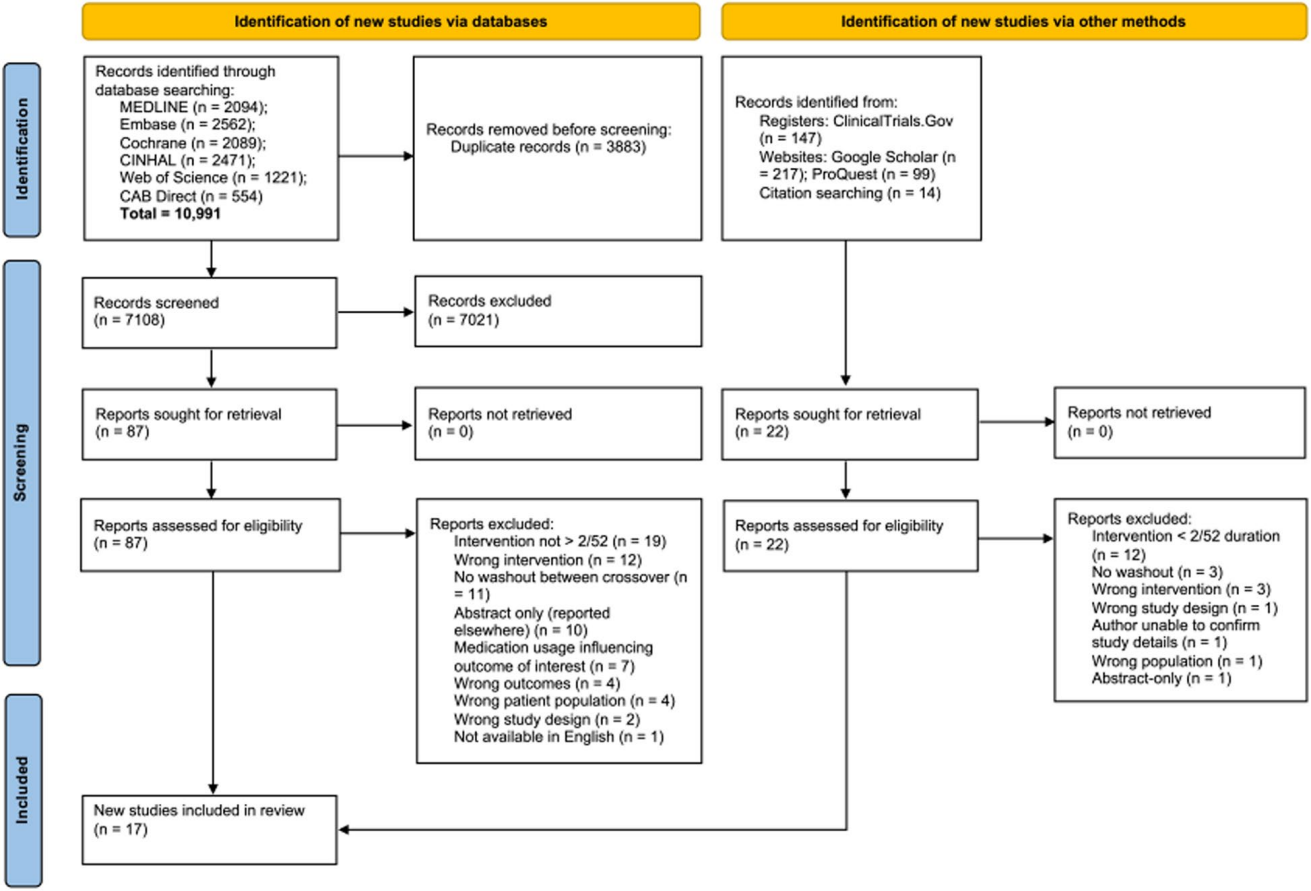
PRISMA flowchart

Our search yielded 10,991 records from database searches. After removing duplicates, we screened 7108 records. Grey literature searches and bibliography searches of the included records led us to review an additional 377 records. We screened 109 records in full-text to determine eligibility. Of these, 17 published papers based on 16 trials met our inclusion criteria. The results of the search are reported in the PRISMA flowchart (Fig. 1).

Primary reasons for excluding reports were: interventions lasting fewer than two weeks; no washout between interventions in crossover trials; and using an intervention that did not include a specific eating frequency allocation. A list of the excluded studies with reasons can be found in Supplementary file 3.

### Characteristics of included studies

A summary of the eligible studies is shown in Table 2 (a larger summary of the data extraction Table is in Supplementary file 6). Trials were conducted between 1971 and 2020. Trials ranged from two-weeks to one-year in duration and included between 7 and 140 participants. There were eight crossover trials and eight parallel-group trials. Seven trials took place in North America, three in Europe, two in Iran and one in Brazil.

**Table 2:** Characteristics of included trials

First Author/Year	Design	Duration	Sample size	Age, years (Mean, S.D., Range)	Sex	Population	Setting	Inclusion/Exclusion	Meals (n)	Eucaloric OR equicalorific	Energy deficit/balance/surplus
Finkelstein—1971 [30]	Parallel trial	2 × 30 day	8	20–22	Female	Overweight—no defined baseline	Mixed	No exclusions reported	4 vs 6	equicalorific—1700 then 1400 for kcals 1st and 2nd 30 days	Unclear* (likely deficit)*
Young—1971 [31]	Crossover trial	2 × 35 days	12	22.2 (20–25)	Male	Overweight—no baselines	Free-living	No exclusions reported	1 vs 3 vs 6	equicalorific—1800kcals	Deficit
Jenkins – 1989 [32]	Crossover trial	2 × 14 days	7	31–51 / 39.6	Male	Normal—not specified how this was calculated (98–121% of *normal*)	Mixed	No exclusions reported	3 vs 17	eucaloric—Mean 2730 ± 189 kcals	Balanced
Verboeket – 1993 [33]	Parallel trial	28 days	14	46.1 ± 3.3 (20–58)	Female	Overweight and Obese (BMI 30.2 ± 0.8 kg/m2)	Free living	Inclusion: Age 20–60; BMI 25–35 kg/m2 NO clear exclusion criteria	2 vs 4	equicalorific—1000 kcal MAX	Deficit
Murphy – 1996 [34]	Crossover trial	2 × 14 days	11	21–24	Female	Normal and overweight (BMI 23.6 ave; 20.6–27.2 range)	Free living	No exclusions reported	2 vs 12	equicalorific—2000kcals—not measured to activity or BMR	Unclear
Rashidi – 2003 [35]	Crossover trial	2 × 14 days	15	27.2 ± 6.4	Male	Unclear. Reported mean weight of 66.8 ± 11.1	Free living	No exclusions reported	3 vs 9	Unclear	Unclear
Stote [36] Carlson – 2007 [37]	Crossover trial	2 × 56 days	21	45 ± 0.7 (40–50)	Both (10 female completers)	Normal	Mixed	Exclusions: smoking, pregnancy or lactation, history of Cardiovascular Disease (CVD) or medication use for CVD, Hypertension (HTN), Diabetes Mellitus (DM), psychiatric disorders, cancer, or high-risk occupations	1 vs 3	Eucaloric	Balance
Berteus-Forslund [38]	Parallel trial	365 days	140	Each group. 40.6 ± 11.1 and 41.8 ± 11.0	Both (27 male:66 female)	Obese	Free living	Inclusions: Age 18–60, BMI > 30. Exclusions: history of obesity surgery, anti-obesity drug treatment within a year; drug or insulin dependent diabetes; hypothyroidism; severe psychiatric disorder; bulimia; drug or alcohol abuse	3 vs 6	Eucaloric	Energy balance—min 1400kcals
Cameron [39]	Parallel trial	56 days	18	18–55	Both	Obese (BMI 30–45)	Free living	Inclusion: Obese, non-diabetic, non smokers, non pregnant, sedentary, weight stable > 6/12, Pre-menopausal	3 vs 6	Groups were prescribed a 700 kcal deficit. Neither equicalorific or eucaloric	Deficit
Bachman [40]	Parallel trial	182 days	51	21–65	Both	Overweight and obese (BMI 27–45)	Free living	Inclusion: Age, BMI. Exclusions: heart or chest condition, or concerns on Physical Activity Readiness Questionnaire (PAR-Q). Unable to walk 2 blocks. Psychiatric illness. DM type 1 or 2. Recent weight change or participation in weight loss program. Intention to leave Tennessee. Pregnant or Lactating or < 6/12 post partum. Consent withheld for group meetings or diary keeping	3 vs 6	Unclear—all running different kcal deficit—'Grazing' intervention averages 110 kcals higher overall	Deficit—either 1200 or 1500 kcals based upon body weight
Arciero [41]	Parallel trial	56 days	30	46 ± 11/ 47 ± 9/ 45 ± 9 (45.9—9.4)	Both (4 male and 24 female)	Overweight and obese (BMI 30.3 ± 5.9)	Free living	Inclusions: Non-smoker; healthy men/women with no cardio or metabolic disorder; inactive < 30 min over 2/7 of structured physical activity, overweight or obese, middle-aged and weight stable (within 2 kg past 6/12)	3 vs 3 high-protein vs 6	Eucaloric	Balanced—RMR and PAL taken
Hatami – 2014 [42]	Parallel trial	90 days	90	36.4 ± 9.7	Both (72 – 80%—female)	Overweight/Obese—INT (mean BMI 30.9 ± 5.1): CON (mean BMI 30.3 ± 4.7)	Free living	Inclusion criteria: Age 20–60, not on cholesterol lowering medication, not on lipid-lowering or weight-controlled diet, free from potential causes of secondary hypercholesterolaemia (e.g. pregnancy, or hyperthyroidism), not on night shifts, non-smoker, and no chronic diseases (heart, kidney or cancer)	6 vs 5	Eucaloric	Deficit—given 400 kcals less than calculated daily requirement
Alencar (Kulovitz) [43]	Crossover trial	2 × 14 days	11	52 ± 7	Female	Obese	Free living	Inclusions: nondiabetic; sedentary to moderately active—30 min per day; weight stable; less that 4 meals/snacks per day habitually Exclusions: medications (diuretics); supplements including caffeine; pacemaker; smoker; bodyweight loss > 3 kg in 6/12; bariatric surgery; changed physical activity; pregnant or lactating; DM/thyroid/cancer/gastrointestinal issues of absorption/liver or kidney disease; allergies; unstable medications e.g. statin status; alcohol consumption	2 vs 6	Equicalorific 1200kcals p/day	Deficit
Perrigue [44]	Crossover trial	2 × 21 days	15	28.5 ± 8.7	Both (4 male:11 female)	Normal and overweight (BMI 23.3 ± 3.4)	Free living	Exclusion criteria: diabetes, smoking, dieting, athletes in training, abnormal blood cholesterol or blood pressure values, medication other than oral contraceptives, pregnant or lactating females	3 vs 8	Eucaloric—not reported how calculated	Balanced
Hagele – 2018 [45]	Crossover trial	2 × 14 days	26	24.7 ± 3.2	Both 13 women:13 men	Normal and overweight (BMI range 19.1—33.3 kg/m2)	Free living	Exclusion criteria: daily consumption of fruit juice or sugar-sweetened beverages outside of the trial, fructose intolerance, habitual meal skipping, chronic diseases, regular use of medication or supplements, alternative eating habits, or smoking	3 vs 6	Unclear—orange juice consumption was calibrated to 20% of daily energy requirements but 3 meals in both groups were uncontrolled	Unclear
Grangiero – 2021 [46]	Parallel trial	90 days	47	29.1 ± 9.2	Female	Class I&II obese (BMI between 30–39.9)	Free living	Exclusion criteria: smokers, menopausal age, not dieting or had a recent change in weight-status, history of bariatric surgery, athletes, medication or supplements for weight control, acute or chronic kidney disease, dyslipidemia, illiteracy, severe food compulsion measured via the Binge Eating Scale	3 vs 6	Eucaloric based on individualized measurements to attain -700 kcal balance	Deficit

Seventeen reports covered 16 distinct trials comparing the effect of meal frequency. Five trials included only female participants, three included only male participants, and eight included participants of both sexes. Thirteen of the trials selected from people considered as obese at the start of the trial, and three selected only those considered as ‘normal’ weight. Three trials provided some or all meals within a controlled environment; 13 trials allowed participants to consume their meals ‘free-living’.

Five studies provided diets that were equicalorific (where participants all receive the same amount of kcals regardless of energy output), seven aimed for eucaloric intakes (where kcals provided by meals attempt to match the amount given to the participant’s basal metabolic rate and their energy expenditure) and four studies could not be classified as either equicalorific or eucaloric. Seven trials aimed for a total kcal deficit on the prescribed meal frequencies, five trials aimed for balanced kcal intakes and in four trials it was unclear.

Of the 16 trials two lasted longer than 100-days, both used a parallel trial design [38, 40]. These two trials by Berteus-Forslund et al. (2008) and Bachman et al. (2012) recruited obese or overweight patients and both utilized a three vs six meal comparator. Berteus-Forslund suffered a high drop out rate (140 participants were randomized, with 93 completing the respective interventions). Berteus-Forslund et al. reported a significant elevation of HDL cholesterol favoring the low frequency (three-meal per day) group, whereas Bachman et al. reported no differences in the high or low frequency eating groups for BMI, fat-free mass, or body fat %. Both groups lost equal amounts of weight overall having been assigned calorie deficit diets over their respective six and 12-month interventions.

### Risk of bias

Our risk of bias assessment is shown in Table 3.

**Table 3:**
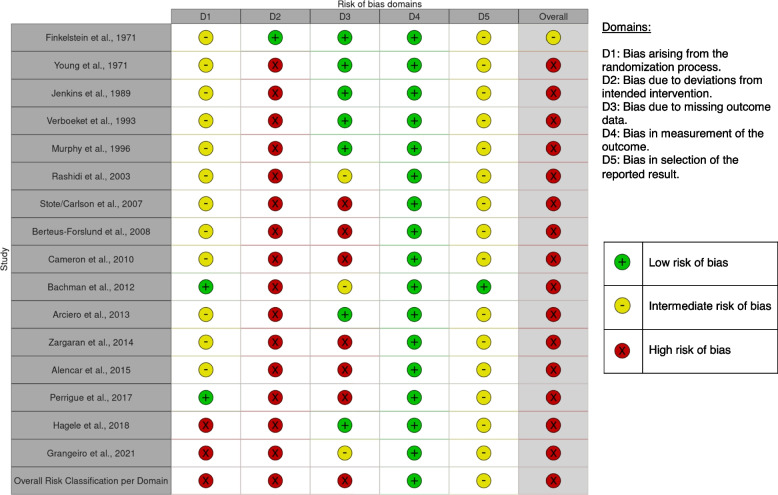
Risk of bias assessment

Two trials reported methods of randomisation [40, 44]. 12 trials were unclear about how participants were randomised. In two cases randomisation was not mentioned and therefore we rated the studies at high-risk of bias [45, 46].

Fifteen trials were at high risk of bias due to potential deviations from the intended intervention. Finklestein et al. [30], demonstrated high-fidelity (no deviations) as they maintained tight control over all the daily meals participants consumed in a laboratory setting. All other trials reported different compliance rates between intervention groups, or had deviations from the intervention that may have affected the outcome. None of the trials attempted to analyse the effect of adherence to the intervention.

Six trials had significant loss to follow-up with their participants, or did not use intention-to-treat analysis—we judged the trials as high risk of bias for missing outcome data [36, 38, 39, 42–44]. Seven trials did not lose any participants to follow-up and therefore we rated them at low-risk for missing outcome data.

Fifteen trials had no publicly available protocol to assess reported outcomes against those planned a priori. Therefore, we judged the trials as at some concern for bias.

We judged 15 trials as at ‘high risk’ in at least one bias domain. Fifteen were judged as at high-risk of bias at the trial level. Domains 1–3 had at least two studies demonstrating a high-risk of bias, and therefore were considered to be at high-risk of bias at the domain-level. Domain 4 – measurement of the outcome was the only domain considered at low-risk of bias at the domain level.

### Results of synthesis

Five trials were only included in the qualitative synthesis. Three trials [30, 36, 42] compared eating frequencies that were either both restricted or both unrestricted, and were excluded from meta-analysis. One trial [35] reported pre-trial values for weight but no post-values, and post-values for insulin, triglycerides and cholesterol but no pre-trial values. We contacted the lead author who was unable to provide the information.

### Outcomes

No trials assessed blood sugar control using HbA1c. Excluding data from cross-over trials from the primary meta-analysis (weight change) did not substantially change the effect estimate. Therefore, we combined data from all available trials in the meta-analyses. No sensitivity analysis was conducted based on trial-level risk of bias because all trials were at high overall risk of bias. A funnel plot was generated for the primary outcome (weight change) but may not convey the full picture of small trial bias given there were only eight trials included in the funnel plot (Supplementary file 4).

Our GRADE assessment of the certainty of the evidence has been integrated within each of the outcomes, the full GRADE Table with explanatory notes can be found in Supplementary file 5.

### Weight change

Eight trials (*n* = 279 participants) assessed the effect of low vs high eating frequencies on weight change. There was very low certainty evidence of no difference between low and high eating frequency on weight change (mean difference: -0.62 kg (95% confidence interval: -2.76 to 1.52 kg, *p* = 0.57, I^2^ = 63%) (Table 4, Supplementary file 5). The populations studied were small; participants were all young to middle-aged adults. Trial populations were a mix of healthy, overweight and obese, but with no reported co-morbidities at baseline. We downgraded the certainty of evidence due to high risk of bias, moderate inconsistency (I^2^ = 63%), indirectness (trials included both equicalorific and eucaloric interventions), and imprecision (fewer than 400 participants, the lack of an agreed effect size estimator, and the wide confidence intervals).

**Table 4:**
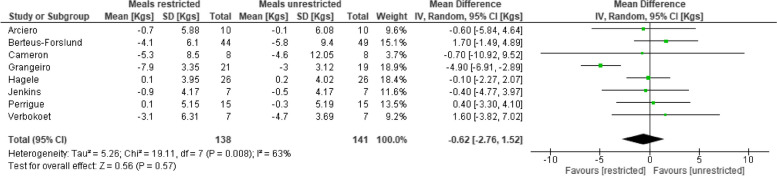
Forest plot comparing low (≤ three meals/day) vs high (≥ four meals/day) meals effect on weight change (kg)

### BMI

Five trials (*n* = 230 participants) assessed the effect of low vs high eating frequencies on BMI. There was very low certainty evidence of no difference between low and high eating frequency on BMI (mean difference: -0.4 kg/m^2^ (95% confidence interval: -0.81 to 0.02 kg/m^2^, *p* = 0.06, I^2^ = 0%) (Table 5, Supplementary 5). The populations studied were small; participants were all young to middle-aged adults. Trial populations were a mix of healthy, overweight and obese, with no reported co-morbidities at baseline. We downgraded the certainty of evidence due to high risk of bias, indirectness (heterogeneity of trial designs), and imprecision (low number of participants, and wide confidence intervals).

**Table 5:**
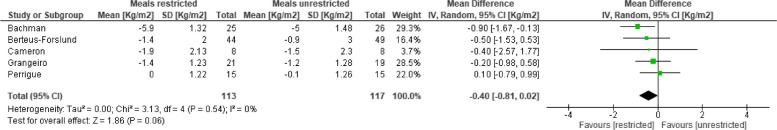
Forest plot comparing low (≤ three) vs high (≥ four) meals effect on BMI (kg/m.^2^)

### Fat Mass

Five trials (*n* = 142 participants) assessed the effect of low vs high eating frequencies on Fat mass (kgs). There was very low certainty evidence of no difference between low and high eating frequency on fat mass (standardized mean difference: -0.28kgs (95% confidence interval: -0.62 to -0.05 kg/m^2^, *p* = 0.10, I^2^ = 0%) (Table 6, Supplementary file 5). The populations studied were small; participants were all young to middle-aged adults. Trial populations were a mix of healthy, overweight and obese, with no reported co-morbidities at baseline. We downgraded the certainty of evidence due to high risk of bias across all included studies, indirectness (heterogeneity of trial designs), and imprecision (low number of participants and wide confidence intervals).

**Table 6:**
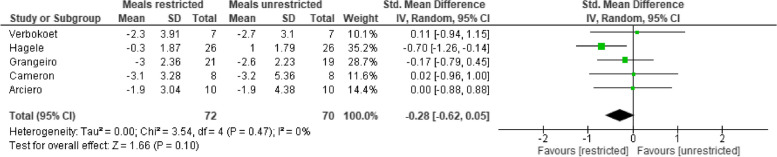
Forest plot comparing low (≤ three) vs high (≥ four) meals effect on fat mass (kg)

### Blood Glucose

Three trials (*n* = 155 participants) assessed the effect of low vs high eating frequencies on blood glucose (mmol/L). There was very low certainty evidence of no difference between low and high eating frequency on blood glucose (mean difference: -0.09 mmol/L (95% confidence interval: -0.23 to 0.05 mmol/L, *p* = 0.21, I^2^ = 42%) (Table 7, Supplementary file 5). The trial populations studied were small; participants were all young to middle-aged adults. All three trials studied obese adults with no reported co-morbidities at baseline. We downgraded the certainty of evidence due to high risk of bias across all trials, inconsistency (large variations in the point estimates and a moderate I^2^ value), indirectness (heterogeneity of trial designs), and imprecision (low number of participants and wide confidence intervals).

**Table 7:**
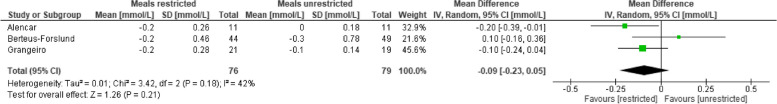
Forest plot comparing low (≤ three) vs high (≥ four) meals effect on blood glucose (mmol/L)

### Insulin

Three trials (*n* = 155 participants) assessed the effect of low vs high eating frequencies on insulin (mU mL-1). There was very low certainty evidence of no difference between low and high eating frequency on blood glucose (mean difference: -3.38 mU mL-1 (95% confidence interval: -6.87 to 0.11 mU mL-1, *p* = 0.06, I^2^ = 67%) (Table 8, Supplementary file 5). The trial populations studied were small; participants were all young to middle-aged adults. All three trials studied obese adults with no reported co-morbidities at baseline. We downgraded the certainty of evidence due to high risk of bias across all trials, indirectness (heterogeneity of trial designs), and imprecision (low number of participants).

**Table 8:**
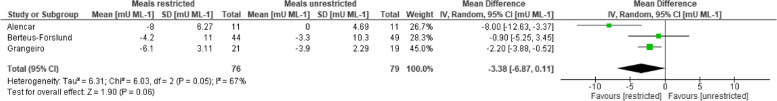
Forest plot comparing low (≤ three) vs high (≥ four) meals effect on insulin (mU mL-1)

### Triglycerides

Six trials (*n* = 243 participants) assessed the effect of low vs high eating frequencies on triglycerides (mmol/L). There was very low certainty evidence of no difference between low and high eating frequency on triglycerides (mean difference: -0.02 mmol/L (95% confidence interval: -0.08 to 0.05 mmol/L, *p* = 0.28, I^2^ = 20%) (Table 9, Supplementary file 5). The trial populations studied were small; participants were all young to middle-aged adults. Participants were a mixture of obese and healthy weight adults with no comorbidities. We downgraded the certainty of evidence due to high risk of bias across all trials, indirectness (heterogeneity of trial designs), and imprecision (low number of participants).

**Table 9:**
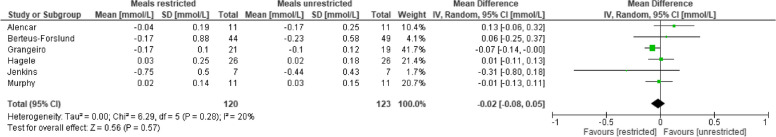
Forest plot comparing low (≤ three) vs high (≥ four) meals effect on triglycerides (mmol/L)

### Total cholesterol

Five trials (*n* = 191 participants) assessed the effect of low vs high eating frequencies on total cholesterol (mmol/L). There was very low certainty evidence of no difference between low and high eating frequency on total cholesterol (mean difference: 0.09 mmol/L (95% confidence interval: -0.12 to 0.29 mmol/L, *p* = 0.41, I^2^ = 63%) (Table 10, Supplementary file 5). The trial populations studied were small; participants were all young to middle-aged adults. Participants were a mixture of healthy weight, overweight, and obese adults with no comorbidities. We downgraded the certainty of evidence due to high risk of bias across all trials, moderate inconsistency (I^2^ – 63%), indirectness (heterogeneity of trial designs), and imprecision (low number of participants, and wide confidence intervals).

**Table 10:**
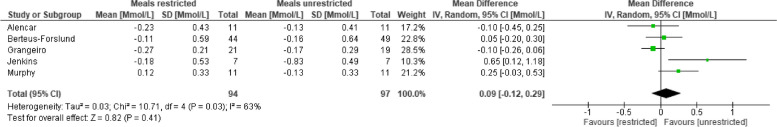
Forest plot comparing low (≤ three) vs high (≥ four) meals effect on total cholesterol (mmol/L)

### LDL cholesterol

Five trials (*n* = 191 participants) assessed the effect of low vs high eating frequencies on LDL cholesterol (mmol/L). There was very low certainty evidence of no difference between low and high eating frequency on LDL cholesterol (mean difference: 0.05 mmol/L (95% confidence interval: -0.13 to 0.23 mmol/L, *p* = 0.62, I^2^ = 59%) (Table 11, Supplementary file 5). The trial populations studied were small; participants were all young to middle-aged adults. Participants were a mixture of healthy weight, overweight, and obese adults with no comorbidities. We downgraded the certainty of evidence due to high risk of bias across all trials, indirectness (heterogeneity of trial designs), and imprecision (low number of participants and wide confidence intervals).

**Table 11:**
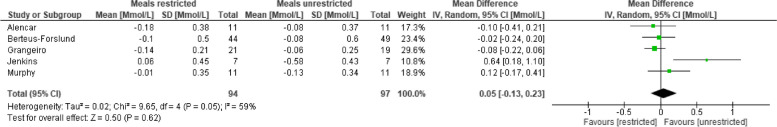
Forest plot comparing low (≤ three) vs high (≥ four) meals effect on LDL (mmol/L)

### HDL

Five trials (*n* = 191 participants) assessed the effect of low vs high eating frequencies on HDL cholesterol (mmol/L). There was very low certainty evidence of no difference between low and high eating frequency on HDL cholesterol (mean difference: 0.05 mmol/L (95% confidence interval: -0.00 to 0.09 mmol/L, *p* = 0.07, I^2^ = 22%) (Table 12, Supplementary file 5). The trial populations studied were small; participants were all young to middle-aged adults. Participants were a mixture of healthy weight, overweight, and obese adults with no comorbidities. We downgraded the certainty of evidence due to high risk of bias across all studies, indirectness (heterogeneity of trial designs), and imprecision (low number of participants).

**Table 12:**
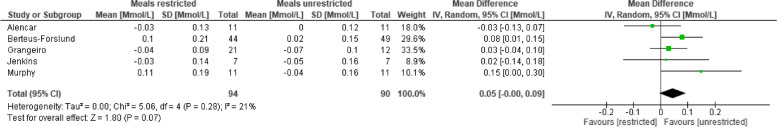
Forest plot comparing low (≤ three) vs high (≥ four) meals effect on HDL (mmol/L)

### Subgroup and sensitivity analyses

Due to insufficient data, none of our planned subgroup analyses were conducted. In an alteration to our protocol we did conduct one sensitivity analysis to assess whether trials that included drinks-alone as meals affected our findings. The removal of the single trial that counted drinks as a meal (Hagele et al., 2018) did not change our results for weight change, fat-mass, or triglycerides. All results remained neutral favouring neither high- or low-meal frequency (See supplementary file 7 for point estimates).

## Discussion

In this meta-analysis of different eating frequencies in clinical trials (parallel or crossover) that last a minimum of two-weeks, we found no difference between eating high- or low-meal frequency on cardiometabolic health. The majority of trials that compare eating frequency had small sample sizes, and did not report the minimum sample size to detect statistical or clinically-significant difference between groups.

Of the 16 trials in our review, Finkelstein et al.’s 1971 trial [30] was the most tightly controlled; participants were observed while they ate in the laboratory. Although 15 of the 16 trials were at high risk of bias, we did not rate any down due to lack of blinding of participants and researchers, as we did not believe this likely affected measurements of the outcome given the tightly controlled procedures for collecting weight measurements and blood draws.

Previous systematic reviews on the health effects of eating frequency had at least six limitations. The authors of those reviews:

1. drew conclusions based primarily on animal studies
2. included trials that were too short to infer a change in cardiometabolic variables (i.e. < two weeks)
3. included parallel or crossover trials with no clear washout period between interventions
4. included studies with research designs that did not address the question of causality/difference in interventions
5. focused on performance rather than health metrics e.g. body composition;
6. recruited populations that limit the generalizability of study findings (e.g. athletes in heavy training) [21, 23, 47, 48].

From a 2021 systematic review that addressed the question of how portion size and eating (‘ingestive’) frequency affected health, authors concluded that there was insufficient information to determine chronic effects on body weight. Those authors only found four papers on eating frequency, none of which met our inclusion criteria [49]. Separate meta-analysis focused only on how meal frequency affected cardiometabolic factors (cholesterol, insulin, blood sugar, and triglycerides) [50]. Several of their included studies did not meet our inclusion criteria due to: (i) the lack of a washout period between crossover interventions, or (ii) the inclusion of patients on medications that affect the outcomes of interest (e.g. bodyweight). In contrast to our conclusions, those authors’ meta-analysis [50] found that unrestricted (high) eating frequency was associated with a healthier total cholesterol and LDL-cholesterol profile.

Between-group results for BMI, Fat Mass and Insulin (see Tables 6, 7 and 8) were not statistically significantly different, however all of them showed trends that favored healthier outcomes for the groups eating less frequently. Despite this trend in the pooled estimates, the very low certainty of our results limit our ability to conclude strongly in favor of either intervention. Therefore, we conclude there was no superiority of either meal frequency on any of these outcomes.

We defend our choice to include studies of at least two weeks’ duration. Past studies of shorter durations have demonstrated that a change in frequency creates a short-term disturbance in homeostasis resulting in examples such as lowered cholesterol and triglycerides which return to the individuals normal homeostatic levels once the body adapts to the intervention [51].

### Limitations of our systematic review

Our meta-analysis for our primary outcome, weight change, included 279 participants. This means the meta-analysis is underpowered—the Cochrane Collaboration recommends a minimum of 400 participants to gain a good level of precision in the absence of known effect estimates [28]. We excluded many trials as a result of cross-over studies not including a wash-out period [43, 51–60], leading to concerns of carry-over effects between interventions. None of the crossover trials in our systematic review assessed their interventions for carry-over effects from the initial interventions, but all included at least one week as a washout period. There is no agreed minimum wash-out period to exclude the effects of the meal frequency in the first intervention of the study carrying over into the second intervention period.

Four trials had 40 or more participants complete their prescribed interventions. The largest and longest of the studies [38] suffered from a very high dropout rate—a common occurrence in dietary intervention trials [61–63]. Berteus-Forslund et al. [38], also reported that their prescribed meal frequencies converged over the intervention period: rather than the prescribed three meal versus six meal intervention, the observed differences were closer to four versus five meals. Studies of eating frequency often suffer from under-reporting (of eating occasions) when participants are studied in the field, leading to detection bias [14, 64–66]. We did not exclude studies that used solely self-reported data for eating frequency, and therefore we cannot rule this out as a confounding variable in our analyses. Where adherence was tracked across trials, adherence was often reported as < 90% further limiting the ability to observe any true effects of the differences in meal frequency interventions. This was reflected in 15 of the 16 included trials being rated as at high-risk of bias due to likely deviations from the intended intervention.

Definitions of an eating occasion were published formally in 2017 [3]. Much of the research included in our study was published prior to this definition and there has been differences in researchers application of what constitutes a meal. We made a pragmatic decision to define an eating or drinking occasion (meal) as any ingestion of kcals, consumed either by food or beverage, with each single episode lasting no longer than one hour (including all snacks, drinks and other instances of calorific intake) [67]. Due to heterogeneity in defining a ‘meal’ in the trials included in our meta-analysis, we cannot rule out the potential for meals being over or under counted when measured using our definition. The problem of no uniform definition across studies has been identified previously alongside a question about how to classify ‘drink-only’ occasions where some drinks (for example non sugar sweetened coffee or tea) carry negligible impact in terms of energy intake (< 210 kJ) but sugar-sweetened beverages contain large amounts of calories which would contribute significantly towards daily energy intake and likely meet the American Heart Association definition of a meal contributing > 15% of a person’s total daily energy intake [3, 68]. As the debate over whether to ‘count’ drinks—which have the potential to effect physiological and therefore health outcomes—has not been resolved, we decided to include them under our broad definition. To further address the potential influence counting ‘drinks-only’ as meals had on our results, we conducted a sensitivity analysis removing the single trial where drinks were allowed to count as a meal with no change to our results.

The trials we included had heterogenous designs. This variation occurred largely within four categories:

- Large discrepancy in the high-meal frequencies studied (e.g. meal frequencies ranged from four to 17);
- Large variation in intervention length (range 14–365 days);
- Differences in baseline weight status of participants (i.e. normal, obese, severely obese);
- Infrequent accounting for whether total ingested kcals (regardless of meal frequency) resulted in calorie deficit, surplus or balance.

This introduced a high level of imprecision amongst the interventions, reducing both our ability to detect any meaningful differences between frequencies and our confidence in the results of the meta-analysis.

Inability to establish whether trials were targeting energy balance, deficit or surplus could have further impeded our ability to detect any meaningful effects of eating frequency. Alongside heterogeneity in physical activity, all studies recommended restrictions to exercise but did not monitor physical activity. The overall calorie balance is a large covariate in studies of dietary behaviour and needs to be controlled in future studies.

Further potential confounding variables were the lack of information included in trials about macro or micronutrient intakes of eating frequency interventions, and the lack of information on timing of the eating occasions. We acknowledge that different macro and micronutrient intakes in the trials’ diets may have been confounding variables.

### Recommendations for future research

We add our voice to those concerned with the quality and quantity of the literature on eating frequency [69]. The 2017 definition of what constitutes an eating occasion should be adopted across all future studies to ensure consistency in intervention designs [3, 70] However, there have been calls for further research into the definition itself due to the vast implications this has for studying eating behaviours [68].

Eating frequency trials carried out in the field are challenging due to concerns over the legitimacy of self-reported dietary data [21]. We believe the establishment of an effective biomarker to measure an eating occasion will be a leap forwards for studies looking to establish causal claims associated with eating frequency [64]. Researchers may benefit from continuous glucose monitors and wearables—these may help us define an eating occasion, and eliminate self-reporting bias by making objective data easier to capture.

To enhance reporting of eating frequency interventions, we encourage researchers to adopt the Template for Intervention Description and Replication (TIDieR) checklist to enhance transparency and reproducibility of their interventions [71].

We also recommend that future eating frequency trials designed to elucidate the effects of meal frequency on cardiometabolic health include six design elements. They are:

1. Interventions should last a minimum of two weeks
2. To ensure there are no carryover effects of crossover trials there should be a minimum wash out period of one week; statistical methods should also take crossover issues into account
3. Outcome measures should include DEXA for fat mass, HbA1c for glycemic control, and Apolipoprotein B (ApoB) for blood lipids
4. Researchers should be explicit about whether diets provide a kcal deficit, neutral load, or surplus
5. Researchers should report measures of physical activity to separate the effects of eating frequency from total kcal consumption
6. Researchers should measure and report adherence to the intervention, to support interpreting the effectiveness of eating frequency as a behavioural intervention.

## Conclusion

Restricted and unrestricted eating frequency had similar effects on weight change and other markers of cardiometabolic health. Trials of eating frequency interventions have been underpowered and biased—therefore we must conclude there is very low certainty to current conclusions. We make six recommendations for future research into eating frequency which is an easily altered dietary behaviour. These recommendations may support greater consistency and precision of future results, which in turn could inform future dietary guidelines on healthy eating behaviours.

### Supplementary Information

**Additional file 1.** Searches from all databases and grey literature searches.

**Additional file 2. **Search filters.

**Additional file 3.** Excluded studies with reasons.

**Additional file 4.** Funnel plot analysis of eligible trials assessing the impact of meal frequency on weight change.

**Additional file 5.** GRADE assessment table with all explanations.

**Additional file 6.** Long Data Extraction.

**Additional file 7**. Sensitivity analysis excluding trials counted drinks-only as meals

## Data Availability

All data informing the meta-analysis are available on request from the corresponding author either as an Excel or Revman file. The full qualitative data extraction sheet is included in the supplementary material.

## Abbreviations

SMD: Standardized Mean Difference
GRADE: Grading of Recommendations Assessment, Development, and Evaluation
BMI: Body Mass Index
PRISMA: Preferred Reporting Items for Systematic Review and Meta-Analysis
CINAHL (EBSCO): Cumulative Index to Nursing and Allied Health (Elton Bryson Stephens Company)
CENTRAL: Cochrane Central Register of Controlled Trials
HbA1c: Hemoglobin A1c
HDL: High Density Lipoprotein
LDL: Low Density Lipoprotein
BIA: Bioelectrical Impedance Assessment
CVD: Cardiovascular Disease
HTN: Hypertension
DM: Diabetes Mellitus
PAR-Q: Physical Activity Readiness Questionnaire
RMR: Resting Metabolic Rate
PAL: Physical Activity Level
TIDiER: Template for Intervention Description and Replication
DEXA: Dual-Energy X-ray Absorptiometry
ApoB: Apolipoprotein B
