# Supplementary material for: The effects of eating frequency on changes in body composition and cardiometabolic health in adults: a systematic review with meta-analysis of randomized trials
Source: Int J Behav Nutr Phys Act. 2023 Nov 14;20:133. doi: 10.1186/s12966-023-01532-z (PMC10647044; doi:10.1186/s12966-023-01532-z)
Supplement: Supplementary file 1 — Additional file 1. Searches from all databases and grey literature searches. [file 12966_2023_1532_MOESM1_ESM.docx]

**Supplementary file 1**

Searches from all databases and grey literature searches including links to specific search filters where appropriate.

**Embase (2562)**

Embase <1974 to 2022 Jun 7>

| 1 | Meal/ | 20682 |
| --- | --- | --- |
| 2 | feeding behavior/ | 89559 |
| 3 | meal frequency/ | 379 |
| 4 | ((meal or feed* or eat* or ingestive) adj3 (frequency or pattern* or episode* or behavio$r)).tw. | 34550 |
| 5 | (gorging or gorge).tw. | 1061 |
| 6 | (fast* adj2 (protocol or plan or implement* or regimen or diet or prescription or requirement or lifestyle or intermittent)).tw. | 5567 |
| 7 | (snacking or snacks).tw. | 8492 |
| 8 | Adipose Tissue/ | 84660 |
| 9 | adipose tissue.tw. | 105455 |
| 10 | adiposity.tw. | 39072 |
| 11 | body mass/ | 542315 |
| 12 | body mass index.tw. | 311206 |
| 13 | BMI.tw. | 361945 |
| 14 | body weight/ | 339616 |
| 15 | body weight.tw. | 302530 |
| 16 | Body fat.tw. | 47977 |
| 17 | high density lipoprotein cholesterol/ | 121236 |
| 18 | HDL cholesterol.tw. | 43388 |
| 19 | low density lipoprotein cholesterol/ | 119683 |
| 20 | LDL cholesterol.tw. | 38801 |
| 21 | glucose blood level/ | 286663 |
| 22 | blood glucose.tw. | 121914 |
| 23 | diabetes mellitus/ | 625697 |
| 24 | diabetes.tw. | 902380 |
| 25 | hemoglobin A1c/ | 129683 |
| 26 | glycated h?emoglobin A*.tw. | 4152 |
| 27 | hba1c.tw. | 86301 |
| 28 | triacylglycerol/ | 218247 |
| 29 | triglycerides.tw. | 100334 |
| 30 | or/8-29 | 2366022 |
| 31 | (Randomized controlled trial/ or Controlled clinical study/ or random*.ti,ab. or randomization/ or intermethod comparison/ or placebo.ti,ab. or (compare or compared or comparison).ti. or ((evaluated or evaluate or evaluating or assessed or assess) and (compare or compared or comparing or comparison)).ab. or (open adj label).ti,ab. or ((double or single or doubly or singly) adj (blind or blinded or blindly)).ti,ab. or double blind procedure/ or parallel group*1.ti,ab. or (crossover or cross over).ti,ab. or ((assign* or match or matched or allocation) adj5 (alternate or group*1 or intervention*1 or patient*1 or subject*1 or participant*1)).ti,ab. or (assigned or allocated).ti,ab. or (controlled adj7 (study or design or trial)).ti,ab. or (volunteer or volunteers).ti,ab. or human experiment/ or trial.ti.) not (((random* adj sampl* adj7 ("cross section*" or questionnaire*1 or survey* or database*1)).ti,ab. not (comparative study/ or controlled study/ or randomi?ed controlled.ti,ab. or randomly assigned.ti,ab.)) or (Cross-sectional study/ not (randomized controlled trial/ or controlled clinical study/ or controlled study/ or randomi?ed controlled.ti,ab. or control group*1.ti,ab.)) or (((case adj control*) and random*) not randomi?ed controlled).ti,ab. or (Systematic review not (trial or study)).ti. or (nonrandom* not random*).ti,ab. or "Random field*".ti,ab. or (random cluster adj3 sampl*).ti,ab. or ((review.ab. and review.pt.) not trial.ti.) or ("we searched".ab. and (review.ti. or review.pt.)) or "update review".ab. or (databases adj4 searched).ab. or ((rat or rats or mouse or mice or swine or porcine or murine or sheep or lambs or pigs or piglets or rabbit or rabbits or cat or cats or dog or dogs or cattle or bovine or monkey or monkeys or trout or marmoset*1).ti. and animal experiment/) or (Animal experiment/ not (human experiment/ or human/))) | 5115218 |
| 32 | 3 or 4 or 5 or 6 or 7 | 48628 |
| 33 | 30 and 31 and 32 | 4501 |
| 34 | juvenile/ or exp adolescent/ or exp child/ or exp postnatal development/ or (pediatric* or paediatric* or child* or newborn* or congenital* or infan* or baby or babies or neonat* or pre term or preterm* or premature birth or NICU or preschool* or pre school* or kindergarten* or elementary school* or nursery school* or schoolchild* or toddler* or boy or boys or girl* or middle school* or pubescen* or juvenile* or teen* or youth* or high school* or adolesc* or prepubesc* or pre pubesc*).mp. or (child* or adolesc* or pediat* or paediat*).jn. | 5179729 |
| 35 | 33 not 34 | 3220 |
| 36 | exp animal experiment/ or exp animal model/ or exp experimental animal/ or exp transgenic animal/ or exp male animal/ or exp female animal/ or exp juvenile animal/ or animal/ or chordata/ or vertebrate/ or tetrapod/ or exp fish/ or amniote/ or exp amphibia/ or mammal/ or exp reptile/ or exp sauropsid/ or therian/ or exp monotremate/ or placental mammals/ or exp marsupial/ or Euarchontoglires/ or exp Afrotheria/ or exp Boreoeutheria/ or exp Laurasiatheria/ or exp Xenarthra/ or primate/ or exp Dermoptera/ or exp Glires/ or exp Scandentia/ or Haplorhini/ or exp prosimian/ or simian/ or exp tarsiiform/ or Catarrhini/ or exp Platyrrhini/ or ape/ or exp Cercopithecidae/ or hominid/ or exp hylobatidae/ or exp chimpanzee/ or exp gorilla/ or exp orang utan/ or (animal or animals or pisces or fish or fishes or catfish or catfishes or sheatfish or silurus or arius or heteropneustes or clarias or gariepinus or fathead minnow or fathead minnows or pimephales or promelas or cichlidae or trout or trouts or char or chars or salvelinus or salmo or oncorhynchus or guppy or guppies or millionfish or poecilia or goldfish or goldfishes or carassius or auratus or mullet or mullets or mugil or curema or shark or sharks or cod or cods or gadus or morhua or carp or carps or cyprinus or carpio or killifish or eel or eels or anguilla or zander or sander or lucioperca or stizostedion or turbot or turbots or psetta or flatfish or flatfishes or plaice or pleuronectes or platessa or tilapia or tilapias or oreochromis or sarotherodon or common sole or dover sole or solea or zebrafish or zebrafishes or danio or rerio or seabass or dicentrarchus or labrax or morone or lamprey or lampreys or petromyzon or pumpkinseed or pumpkinseeds or lepomis or gibbosus or herring or clupea or harengus or amphibia or amphibian or amphibians or anura or salientia or frog or frogs or rana or toad or toads or bufo or xenopus or laevis or bombina or epidalea or calamita or salamander or salamanders or newt or newts or triturus or reptilia or reptile or reptiles or bearded dragon or pogona or vitticeps or iguana or iguanas or lizard or lizards or anguis fragilis or turtle or turtles or snakes or snake or aves or bird or birds or quail or quails or coturnix or bobwhite or colinus or virginianus or poultry or poultries or fowl or fowls or chicken or chickens or gallus or zebra finch or taeniopygia or guttata or canary or canaries or serinus or canaria or parakeet or parakeets or grasskeet or parrot or parrots or psittacine or psittacines or shelduck or tadorna or goose or geese or branta or leucopsis or woodlark or lullula or flycatcher or ficedula or hypoleuca or dove or doves or geopelia or cuneata or duck or ducks or greylag or graylag or anser or harrier or circus pygargus or red knot or great knot or calidris or canutus or godwit or limosa or lapponica or meleagris or gallopavo or jackdaw or corvus or monedula or ruff or philomachus or pugnax or lapwing or peewit or plover or vanellus or swan or cygnus or columbianus or bewickii or gull or chroicocephalus or ridibundus or albifrons or great tit or parus or aythya or fuligula or streptopelia or risoria or spoonbill or platalea or leucorodia or blackbird or turdus or merula or blue tit or cyanistes or pigeon or pigeons or columba or pintail or anas or starling or sturnus or owl or athene noctua or pochard or ferina or cockatiel or nymphicus or hollandicus or skylark or alauda or tern or sterna or teal or crecca or oystercatcher or haematopus or ostralegus or shrew or shrews or sorex or araneus or crocidura or russula or european mole or talpa or chiroptera or bat or bats or eptesicus or serotinus or myotis or dasycneme or daubentonii or pipistrelle or pipistrellus or cat or cats or felis or catus or feline or dog or dogs or canis or canine or canines or otter or otters or lutra or badger or badgers or meles or fitchew or fitch or foumart or foulmart or ferrets or ferret or polecat or polecats or mustela or putorius or weasel or weasels or fox or foxes or vulpes or common seal or phoca or vitulina or grey seal or halichoerus or horse or horses or equus or equine or equidae or donkey or donkeys or mule or mules or pig or pigs or swine or swines or hog or hogs or boar or boars or porcine or piglet or piglets or sus or scrofa or llama or llamas or lama or glama or deer or deers or cervus or elaphus or cow or cows or bos taurus or bos indicus or bovine or bull or bulls or cattle or bison or bisons or sheep or sheeps or ovis aries or ovine or lamb or lambs or mouflon or mouflons or goat or goats or capra or caprine or chamois or rupicapra or leporidae or lagomorpha or lagomorph or rabbit or rabbits or oryctolagus or cuniculus or laprine or hares or lepus or rodentia or rodent or rodents or murinae or mouse or mice or mus or musculus or murine or woodmouse or apodemus or rat or rats or rattus or norvegicus or guinea pig or guinea pigs or cavia or porcellus or hamster or hamsters or mesocricetus or cricetulus or cricetus or gerbil or gerbils or jird or jirds or meriones or unguiculatus or jerboa or jerboas or jaculus or chinchilla or chinchillas or beaver or beavers or castor fiber or castor canadensis or sciuridae or squirrel or squirrels or sciurus or chipmunk or chipmunks or marmot or marmots or marmota or suslik or susliks or spermophilus or cynomys or cottonrat or cottonrats or sigmodon or vole or voles or microtus or myodes or glareolus or primate or primates or prosimian or prosimians or lemur or lemurs or lemuridae or loris or bush baby or bush babies or bushbaby or bushbabies or galago or galagos or anthropoidea or anthropoids or simian or simians or monkey or monkeys or marmoset or marmosets or callithrix or cebuella or tamarin or tamarins or saguinus or leontopithecus or squirrel monkey or squirrel monkeys or saimiri or night monkey or night monkeys or owl monkey or owl monkeys or douroucoulis or aotus or spider monkey or spider monkeys or ateles or baboon or baboons or papio or rhesus monkey or macaque or macaca or mulatta or cynomolgus or fascicularis or green monkey or green monkeys or chlorocebus or vervet or vervets or pygerythrus or hominoidea or ape or apes or hylobatidae or gibbon or gibbons or siamang or siamangs or nomascus or symphalangus or hominidae or orangutan or orangutans or pongo or chimpanzee or chimpanzees or pan troglodytes or bonobo or bonobos or pan paniscus or gorilla or gorillas or troglodytes).ti,ab. | 7634190 |
| 37 | 35 not 36 | 2611 |
| 38 | (Pregnancy or Gestational or Child or Congenital or infant or adolescent or pediatrics or child or (pediatric* or paediatric* or child* or newborn* or congenital* or infan* or baby or babies or neonat* or pre-term or preterm* or premature birth* or NICU or preschool* or pre-school* or kindergarten* or kindergarden* or "elementary school*" or nursery school* or (day care* not adult*) or schoolchild* or toddler* or boy or boys or girl* or middle school* or pubescen* or juvenile* or teen* or youth* or high school* or adolesc* or pre-pubesc* or prepubesc*)).mp. [mp=title, abstract, heading word, drug trade name, original title, device manufacturer, drug manufacturer, device trade name, keyword heading word, floating subheading word, candidate term word] | 5679176 |
| 39 | 37 not 38 | 2562 |

**MEDLINE (2094)**

Ovid MEDLINE(R) and Epub Ahead of Print, In-Process, In-Data-Review & Other Non-Indexed Citations, Daily and Versions <1946 to June 7 2022>

| 1 | Meals/ | 4571 |
| --- | --- | --- |
| 2 | Feeding Behavior/ | 91046 |
| 3 | ((meal or feed* or eat* or ingestive) adj3 (frequency or pattern* or episode* or behavio$r)).tw. | 29649 |
| 4 | (gorging or gorge).tw. | 1004 |
| 5 | (fast* adj2 (protocol or plan or implement* or regimen or diet or prescription or requirement or lifestyle or intermittent)).tw. | 4268 |
| 6 | snacking.tw. | 1402 |
| 7 | or/1-6 | 117599 |
| 8 | Adipose Tissue/ | 86720 |
| 9 | adipose tissue.tw. | 80389 |
| 10 | adiposity.tw. | 28332 |
| 11 | Body Mass Index/ | 145087 |
| 12 | body mass index.tw. | 219113 |
| 13 | BMI.tw. | 176955 |
| 14 | Body Weight/ | 197267 |
| 15 | body weight.tw. | 226141 |
| 16 | Body fat.tw. | 36006 |
| 17 | Cholesterol, HDL/ | 30580 |
| 18 | HDL cholesterol.tw. | 29859 |
| 19 | Cholesterol, LDL/ | 30549 |
| 20 | LDL cholesterol.tw. | 25911 |
| 21 | Blood Glucose/ | 178917 |
| 22 | blood glucose.tw. | 81368 |
| 23 | Diabetes Mellitus/ | 132797 |
| 24 | diabetes.tw. | 604840 |
| 25 | Glycated Hemoglobin A/ | 40898 |
| 26 | glycated h?emoglobin A*.tw. | 3186 |
| 27 | hba1c.tw. | 42192 |
| 28 | Triglycerides/ | 80813 |
| 29 | triglycerides.tw. | 69664 |
| 30 | or/8-29 | 1517067 |
| 31 | randomized controlled trial.pt. | 576616 |
| 32 | controlled clinical trial.pt. | 95018 |
| 33 | randomized.ab. | 575190 |
| 34 | clinical trials as topic.sh. | 200352 |
| 35 | randomly.ab. | 390966 |
| 36 | trial.ti. | 269997 |
| 37 | or/31-36 | 1422665 |
| 38 | 7 and 30 and 37 | 4131 |
| 39 | limit 38 to english language | 4048 |
| 40 | exp Child/ or "Congenital, Hereditary, and Neonatal Diseases and Abnormalities"/ or exp infant/ or adolescent/ or exp pediatrics/ or child, abandoned/ or exp child, exceptional/ or child, orphaned/ or child, unwanted/ or minors/ or (pediatric* or paediatric* or child* or newborn* or congenital* or infan* or baby or babies or neonat* or pre-term or preterm* or premature birth* or NICU or preschool* or pre-school* or kindergarten* or kindergarden* or elementary school* or nursery school* or (day care* not adult*) or schoolchild* or toddler* or boy or boys or girl* or middle school* or pubescen* or juvenile* or teen* or youth* or high school* or adolesc* or pre-pubesc* or prepubesc*).mp. or (child* or adolesc* or pediat* or paediat*).jn. | 4990498 |
| 41 | 39 not 40 | 2717 |
| 42 | exp Pregnancy Complications, Infectious/ or exp Pregnancy/ or exp Pregnant Women/ or exp Labor, Obstetric/ or exp Delivery, Obstetric/ or exp Obstetric Labor, Premature/ or exp Obstetric Labor Complications/ or Prenatal Care/ or (obstetric* or prenatal* or pregnan* or gestat* or perinatal or antenatal or parturi* or childbirth or (labo?r adj3 delivery)).mp. | 1291554 |
| 43 | 41 not 42 | 2648 |
| 44 | (exp Animals/ not (exp Animals/ and Humans/)) or ("animal stud*" or "laboratory animal*" or "animal research" or wildlife or pisces or fish or fishes or catfish or catfishes or sheatfish or silurus or arius or heteropneustes or clarias or gariepinus or fathead minnow or fathead minnows or pimephales or promelas or cichlidae or trout or trouts or char or chars or salvelinus or salmo or oncorhynchus or guppy or guppies or millionfish or poecilia or goldfish or goldfishes or carassius or auratus or mullet or mullets or mugil or curema or shark or sharks or cod or cods or gadus or morhua or carp or carps or cyprinus or carpio or killifish or eel or eels or anguilla or zander or sander or lucioperca or stizostedion or turbot or turbots or psetta or flatfish or flatfishes or plaice or pleuronectes or platessa or tilapia or tilapias or oreochromis or sarotherodon or common sole or dover sole or solea or zebrafish or zebrafishes or danio or rerio or seabass or dicentrarchus or labrax or morone or lamprey or lampreys or petromyzon or pumpkinseed or pumpkinseeds or lepomis or gibbosus or herring or clupea or harengus or amphibia or amphibian or amphibians or anura or salientia or frog or frogs or rana or toad or toads or bufo or xenopus or laevis or bombina or epidalea or calamita or salamander or salamanders or newt or newts or triturus or reptilia or reptile or reptiles or bearded dragon or pogona or vitticeps or iguana or iguanas or lizard or lizards or anguis fragilis or turtle or turtles or snakes or snake or aves or bird or birds or quail or quails or coturnix or bobwhite or colinus or virginianus or poultry or poultries or fowl or fowls or chicken or chickens or gallus or zebra finch or taeniopygia or guttata or canary or canaries or serinus or canaria or parakeet or parakeets or grasskeet or parrot or parrots or psittacine or psittacines or shelduck or tadorna or goose or geese or branta or leucopsis or woodlark or lullula or flycatcher or ficedula or hypoleuca or dove or doves or geopelia or cuneata or duck or ducks or greylag or graylag or anser or harrier or circus pygargus or red knot or great knot or calidris or canutus or godwit or limosa or lapponica or meleagris or gallopavo or jackdaw or corvus or monedula or ruff or philomachus or pugnax or lapwing or peewit or plover or vanellus or swan or cygnus or columbianus or bewickii or gull or chroicocephalus or ridibundus or albifrons or great tit or parus or aythya or fuligula or streptopelia or risoria or spoonbill or platalea or leucorodia or blackbird or turdus or merula or blue tit or cyanistes or pigeon or pigeons or columba or pintail or anas or starling or sturnus or owl or owls or athene noctua or pochard or ferina or cockatiel or nymphicus or hollandicus or skylark or alauda or tern or sterna or teal or crecca or oystercatcher or haematopus or ostralegus or sparrow* or shrew or shrews or sorex or araneus or crocidura or russula or european mole or talpa or chiroptera or bat or bats or eptesicus or serotinus or myotis or dasycneme or daubentonii or pipistrelle or pipistrellus or cat or cats or felis or catus or feline or lion or lions or tiger or tigers or cheetah* or jaguar* or lynx* or cougar* or dog or dogs or canis or canine or canines or wolf or wolves or dingo or hyena* or otter or otters or lutra or badger or badgers or meles or fitchew or fitch or foumart or foulmart or ferrets or ferret or polecat or polecats or mustela or putorius or weasel or weasels or fox or foxes or vulpes or wolverine* or skunk* or pika or groundhog* or lemming or muskrat or common seal or dolphin* or "sea cow" or whale or whales or dugong or "sea lion*" or sirenia* or phoca or vitulina or grey seal or halichoerus or horse or horses or equus or equine or equidae or donkey or donkeys or mule or mules or zebra* or pig or pigs or swine or swines or hog or hogs or boar or boars or porcine or piglet or piglets or sus or bear or bears or porcupine* or scrofa or llama or llamas or lama or glama or deer or deers or reindeer* or wapiti or elk or caribou or moose or cervus or elaphus or cow or cows or bos taurus or bos indicus or bovine or bull or bulls or cattle or bison or bisons or sheep or sheeps or ovis aries or ovine or lamb or lambs or mouflon or mouflons or goat or goats or capra or caprine or chamois or rupicapra or leporidae or lagomorpha or lagomorph or rabbit or rabbits or oryctolagus or cuniculus or laprine or hares or lepus or rodentia or rodent or rodents or murinae or mouse or mice or mus or musculus or murine or woodmouse or apodemus or rat or rats or rattus or norvegicus or guinea pig or guinea pigs or cavia or porcellus or hamster or hamsters or mesocricetus or cricetulus or cricetus or gerbil or gerbils or jird or jirds or meriones or unguiculatus or jerboa or jerboas or jaculus or chinchilla or chinchillas or beaver or beavers or castor fiber or castor canadensis or sciuridae or squirrel or squirrels or sciurus or chipmunk or chipmunks or marmot or marmots or marmota or suslik or susliks or spermophilus or cynomys or cottonrat or cottonrats or sigmodon or vole or voles or shrews or woodrat* or microtus or myodes or glareolus or primate or primates or prosimian or prosimians or lemur or lemurs or lemuridae or loris or bush baby or bush babies or bushbaby or bushbabies or galago or galagos or anthropoidea or anthropoids or simian or simians or monkey or monkeys or monkies or marmoset or marmosets or callithrix or cebuella or tamarin or tamarins or saguinus or leontopithecus or squirrel monkey or squirrel monkeys or saimiri or douroucoulis or aotus or ateles or baboon or baboons or papio or rhesus monkey or macaque or macaca or mulatta or cynomolgus or fascicularis or green monkey or green monkeys or chlorocebus or vervet or vervets or pygerythrus or hominoidea or ape or apes or hylobatidae or gibbon or gibbons or siamang or siamangs or nomascus or symphalangus or hominidae or orangutan or orangutans or pongo or chimpanzee or chimpanzees or pan troglodytes or bonobo or bonobos or pan paniscus or gorilla or gorillas or troglodytes).ti,ab. | 6654001 |
| 45 | 43 not 44 | 2094 |

**Cochrane (2089, searched on June 7th)**

EBM Reviews - Cochrane Central Register of Controlled Trials <May 2022>

| 1 | Meals/ | 897 |
| --- | --- | --- |
| 2 | Feeding Behavior/ | 3540 |
| 3 | ((meal or feed* or eat* or ingestive) adj3 (frequency or pattern* or episode* or behavio$r)).tw. | 3216 |
| 4 | (gorging or gorge).tw. | 21 |
| 5 | (fast* adj2 (protocol or plan or implement* or regimen or diet or prescription or requirement or lifestyle or intermittent)).tw. | 849 |
| 6 | snacking.tw. | 306 |
| 7 | or/1-6 | 8065 |
| 8 | Adipose Tissue/ | 2054 |
| 9 | adipose tissue.tw. | 3936 |
| 10 | adiposity.tw. | 2666 |
| 11 | Body Mass Index/ | 11038 |
| 12 | body mass index.tw. | 37180 |
| 13 | BMI.tw. | 45973 |
| 14 | Body Weight/ | 8842 |
| 15 | body weight.tw. | 33516 |
| 16 | Body fat.tw. | 7606 |
| 17 | Cholesterol, HDL/ | 3854 |
| 18 | HDL cholesterol.tw. | 6899 |
| 19 | Cholesterol, LDL/ | 4937 |
| 20 | LDL cholesterol.tw. | 8375 |
| 21 | Blood Glucose/ | 17430 |
| 22 | blood glucose.tw. | 19065 |
| 23 | Diabetes Mellitus/ | 10558 |
| 24 | diabetes.tw. | 84516 |
| 25 | Glycated Hemoglobin A/ | 6360 |
| 26 | glycated h?emoglobin A*.tw. | 808 |
| 27 | hba1c.tw. | 20673 |
| 28 | Triglycerides/ | 6603 |
| 29 | triglycerides.tw. | 13661 |
| 30 | or/8-29 | 194722 |
| 31 | randomized controlled trial.pt. | 555171 |
| 32 | controlled clinical trial.pt. | 93140 |
| 33 | randomized.ab. | 632266 |
| 34 | clinical trials as topic.sh. | 33269 |
| 35 | randomly.ab. | 285847 |
| 36 | trial.ti. | 370112 |
| 37 | or/31-36 | 1236813 |
| 38 | 7 and 30 and 37 | 3393 |
| 39 | limit 38 to english language | 3324 |
| 40 | exp Child/ or "Congenital, Hereditary, and Neonatal Diseases and Abnormalities"/ or exp infant/ or adolescent/ or exp pediatrics/ or child, abandoned/ or exp child, exceptional/ or child, orphaned/ or child, unwanted/ or minors/ or (pediatric* or paediatric* or child* or newborn* or congenital* or infan* or baby or babies or neonat* or pre-term or preterm* or premature birth* or NICU or preschool* or pre-school* or kindergarten* or kindergarden* or elementary school* or nursery school* or (day care* not adult*) or schoolchild* or toddler* or boy or boys or girl* or middle school* or pubescen* or juvenile* or teen* or youth* or high school* or adolesc* or pre-pubesc* or prepubesc*).mp. or (child* or adolesc* or pediat* or paediat*).jn. | 345018 |
| 41 | 39 not 40 | 2236 |
| 42 | exp Pregnancy Complications, Infectious/ or exp Pregnancy/ or exp Pregnant Women/ or exp Labor, Obstetric/ or exp Delivery, Obstetric/ or exp Obstetric Labor, Premature/ or exp Obstetric Labor Complications/ or Prenatal Care/ or (obstetric* or prenatal* or pregnan* or gestat* or perinatal or antenatal or parturi* or childbirth or (labo?r adj3 delivery)).mp. | 95055 |
| 43 | 41 not 42 | 2192 |
| 44 | (exp Animals/ not (exp Animals/ and Humans/)) or ("animal stud*" or "laboratory animal*" or "animal research" or wildlife or pisces or fish or fishes or catfish or catfishes or sheatfish or silurus or arius or heteropneustes or clarias or gariepinus or fathead minnow or fathead minnows or pimephales or promelas or cichlidae or trout or trouts or char or chars or salvelinus or salmo or oncorhynchus or guppy or guppies or millionfish or poecilia or goldfish or goldfishes or carassius or auratus or mullet or mullets or mugil or curema or shark or sharks or cod or cods or gadus or morhua or carp or carps or cyprinus or carpio or killifish or eel or eels or anguilla or zander or sander or lucioperca or stizostedion or turbot or turbots or psetta or flatfish or flatfishes or plaice or pleuronectes or platessa or tilapia or tilapias or oreochromis or sarotherodon or common sole or dover sole or solea or zebrafish or zebrafishes or danio or rerio or seabass or dicentrarchus or labrax or morone or lamprey or lampreys or petromyzon or pumpkinseed or pumpkinseeds or lepomis or gibbosus or herring or clupea or harengus or amphibia or amphibian or amphibians or anura or salientia or frog or frogs or rana or toad or toads or bufo or xenopus or laevis or bombina or epidalea or calamita or salamander or salamanders or newt or newts or triturus or reptilia or reptile or reptiles or bearded dragon or pogona or vitticeps or iguana or iguanas or lizard or lizards or anguis fragilis or turtle or turtles or snakes or snake or aves or bird or birds or quail or quails or coturnix or bobwhite or colinus or virginianus or poultry or poultries or fowl or fowls or chicken or chickens or gallus or zebra finch or taeniopygia or guttata or canary or canaries or serinus or canaria or parakeet or parakeets or grasskeet or parrot or parrots or psittacine or psittacines or shelduck or tadorna or goose or geese or branta or leucopsis or woodlark or lullula or flycatcher or ficedula or hypoleuca or dove or doves or geopelia or cuneata or duck or ducks or greylag or graylag or anser or harrier or circus pygargus or red knot or great knot or calidris or canutus or godwit or limosa or lapponica or meleagris or gallopavo or jackdaw or corvus or monedula or ruff or philomachus or pugnax or lapwing or peewit or plover or vanellus or swan or cygnus or columbianus or bewickii or gull or chroicocephalus or ridibundus or albifrons or great tit or parus or aythya or fuligula or streptopelia or risoria or spoonbill or platalea or leucorodia or blackbird or turdus or merula or blue tit or cyanistes or pigeon or pigeons or columba or pintail or anas or starling or sturnus or owl or owls or athene noctua or pochard or ferina or cockatiel or nymphicus or hollandicus or skylark or alauda or tern or sterna or teal or crecca or oystercatcher or haematopus or ostralegus or sparrow* or shrew or shrews or sorex or araneus or crocidura or russula or european mole or talpa or chiroptera or bat or bats or eptesicus or serotinus or myotis or dasycneme or daubentonii or pipistrelle or pipistrellus or cat or cats or felis or catus or feline or lion or lions or tiger or tigers or cheetah* or jaguar* or lynx* or cougar* or dog or dogs or canis or canine or canines or wolf or wolves or dingo or hyena* or otter or otters or lutra or badger or badgers or meles or fitchew or fitch or foumart or foulmart or ferrets or ferret or polecat or polecats or mustela or putorius or weasel or weasels or fox or foxes or vulpes or wolverine* or skunk* or pika or groundhog* or lemming or muskrat or common seal or dolphin* or "sea cow" or whale or whales or dugong or "sea lion*" or sirenia* or phoca or vitulina or grey seal or halichoerus or horse or horses or equus or equine or equidae or donkey or donkeys or mule or mules or zebra* or pig or pigs or swine or swines or hog or hogs or boar or boars or porcine or piglet or piglets or sus or bear or bears or porcupine* or scrofa or llama or llamas or lama or glama or deer or deers or reindeer* or wapiti or elk or caribou or moose or cervus or elaphus or cow or cows or bos taurus or bos indicus or bovine or bull or bulls or cattle or bison or bisons or sheep or sheeps or ovis aries or ovine or lamb or lambs or mouflon or mouflons or goat or goats or capra or caprine or chamois or rupicapra or leporidae or lagomorpha or lagomorph or rabbit or rabbits or oryctolagus or cuniculus or laprine or hares or lepus or rodentia or rodent or rodents or murinae or mouse or mice or mus or musculus or murine or woodmouse or apodemus or rat or rats or rattus or norvegicus or guinea pig or guinea pigs or cavia or porcellus or hamster or hamsters or mesocricetus or cricetulus or cricetus or gerbil or gerbils or jird or jirds or meriones or unguiculatus or jerboa or jerboas or jaculus or chinchilla or chinchillas or beaver or beavers or castor fiber or castor canadensis or sciuridae or squirrel or squirrels or sciurus or chipmunk or chipmunks or marmot or marmots or marmota or suslik or susliks or spermophilus or cynomys or cottonrat or cottonrats or sigmodon or vole or voles or shrews or woodrat* or microtus or myodes or glareolus or primate or primates or prosimian or prosimians or lemur or lemurs or lemuridae or loris or bush baby or bush babies or bushbaby or bushbabies or galago or galagos or anthropoidea or anthropoids or simian or simians or monkey or monkeys or monkies or marmoset or marmosets or callithrix or cebuella or tamarin or tamarins or saguinus or leontopithecus or squirrel monkey or squirrel monkeys or saimiri or douroucoulis or aotus or ateles or baboon or baboons or papio or rhesus monkey or macaque or macaca or mulatta or cynomolgus or fascicularis or green monkey or green monkeys or chlorocebus or vervet or vervets or pygerythrus or hominoidea or ape or apes or hylobatidae or gibbon or gibbons or siamang or siamangs or nomascus or symphalangus or hominidae or orangutan or orangutans or pongo or chimpanzee or chimpanzees or pan troglodytes or bonobo or bonobos or pan paniscus or gorilla or gorillas or troglodytes).ti,ab. | 47533 |
| 45 | 43 not 44 | 2089 |

**CINAHL (2471)**

**Accessibility Information and Tips**

# Print Search History

| **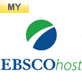** | Tuesday, June 07, 2022 6:02:18 PM |
| --- | --- |

| **#** | **Query** | **Limiters/Expanders** | **Last Run Via** | **Results** |
| --- | --- | --- | --- | --- |
| S45 | S44 NOT S42 | Search modes - Boolean/Phrase | Interface - EBSCOhost Research Databases  Search Screen - Advanced Search  Database - CINAHL Complete | 2,471 |
| S44 | S40 AND S41 AND S43 | Search modes - Boolean/Phrase | Interface - EBSCOhost Research Databases  Search Screen - Advanced Search  Database - CINAHL Complete | 2,594 |
| S43 | S1 OR S2 OR S3 OR S4 OR S5 | Search modes - Boolean/Phrase | Interface - EBSCOhost Research Databases  Search Screen - Advanced Search  Database - CINAHL Complete | 27,762 |
| S42 | TI (pregnan* or parous or primipar* or multipar* or primigravid* or multigravid* or gestation* or gravidit* or parturi* or puerper* or perinatal* or “peri natal*” or prenatal* or “pre natal*” or antenatal* or “ante natal*” or postnatal* or “post natal*” or peripartum or “peri partum” or prepartum or “pre partum” or antepartum or “ante partum” or intrapartum or “intra partum” or postpartum or “post partum” or (expectant* N1 mother*) or “mother-to-be” or “mothers-to-be” or (child N0 bear*) or childbear* or ((before or after or vaginal or abdominal) N1 delivery) or caesarean* or cesarean* or “c-section*” or childbirth or (child* N4 (birth or delivery))) OR AB (pregnan* or parous or primipar* or multipar* or primigravid* or multigravid* or gestation* or gravidit* or parturi* or puerper* or perinatal* or “peri natal*” or prenatal* or “pre natal*” or antenatal* or “ante natal*” or postnatal* or “post natal*” or peripartum or “peri partum” or prepartum or “pre partum” or antepartum or “ante partum” or intrapartum or “intra partum” or postpartum or “post partum” or (expectant* N1 mother*) or “mother-to-be” or “mothers-to-be” or (child N0 bear*) or childbear* or ((before or after or vaginal or abdominal) N1 delivery) or caesarean* or cesarean* or “c-section*” or childbirth or (child* N4 (birth or delivery))) | Search modes - Boolean/Phrase | Interface - EBSCOhost Research Databases  Search Screen - Advanced Search  Database - CINAHL Complete | 281,201 |
| S41 | ( MH ( randomized controlled trials OR double‐blind studies OR single‐blind studies OR random assignment OR pretest‐posttest design OR cluster sample ) OR TI ( randomised OR randomized ) OR AB random* OR TI trial OR ( (MH (sample size) AND AB (assigned OR allocated OR control)) ) OR MH ( placebos OR crossover design OR comparative studies ) OR AB ( (control W5 group) OR (cluster W3 RCT) OR PT (randomized controlled trial)) ) NOT ( ( MH animals+ OR MH (animal studies) OR TI (animal model*) ) NOT MH (human) ) | Search modes - Boolean/Phrase | Interface - EBSCOhost Research Databases  Search Screen - Advanced Search  Database - CINAHL Complete | 918,388 |
| S40 | S6 OR S7 OR S8 OR S9 OR S10 OR S11 OR S12 OR S13 OR S14 OR S15 OR S16 OR S17 OR S18 OR S19 OR S20 OR S21 OR S22 OR S23 OR S24 OR S25 OR S26 OR S27 OR S28 OR S29 OR S30 OR S31 OR S32 OR S33 OR S34 OR S35 OR S36 OR S37 OR S38 OR S39 | Search modes - Boolean/Phrase | Interface - EBSCOhost Research Databases  Search Screen - Advanced Search  Database - CINAHL Complete | 440,507 |
| S39 | AB triglycerides | Search modes - Boolean/Phrase | Interface - EBSCOhost Research Databases  Search Screen - Advanced Search  Database - CINAHL Complete | 20,472 |
| S38 | TI triglycerides | Search modes - Boolean/Phrase | Interface - EBSCOhost Research Databases  Search Screen - Advanced Search  Database - CINAHL Complete | 1,876 |
| S37 | (MH "Triglycerides") | Search modes - Boolean/Phrase | Interface - EBSCOhost Research Databases  Search Screen - Advanced Search  Database - CINAHL Complete | 15,734 |
| S36 | AB hba1c | Search modes - Boolean/Phrase | Interface - EBSCOhost Research Databases  Search Screen - Advanced Search  Database - CINAHL Complete | 13,375 |
| S35 | TI hba1c | Search modes - Boolean/Phrase | Interface - EBSCOhost Research Databases  Search Screen - Advanced Search  Database - CINAHL Complete | 1,792 |
| S34 | AB glycated h#emoglobin A* | Search modes - Boolean/Phrase | Interface - EBSCOhost Research Databases  Search Screen - Advanced Search  Database - CINAHL Complete | 632 |
| S33 | TI glycated h#emoglobin A* | Search modes - Boolean/Phrase | Interface - EBSCOhost Research Databases  Search Screen - Advanced Search  Database - CINAHL Complete | 320 |
| S32 | AB diabetes | Search modes - Boolean/Phrase | Interface - EBSCOhost Research Databases  Search Screen - Advanced Search  Database - CINAHL Complete | 151,477 |
| S31 | TI diabetes | Search modes - Boolean/Phrase | Interface - EBSCOhost Research Databases  Search Screen - Advanced Search  Database - CINAHL Complete | 115,931 |
| S30 | (MH "Diabetes Mellitus") | Search modes - Boolean/Phrase | Interface - EBSCOhost Research Databases  Search Screen - Advanced Search  Database - CINAHL Complete | 72,169 |
| S29 | AB blood glucose | Search modes - Boolean/Phrase | Interface - EBSCOhost Research Databases  Search Screen - Advanced Search  Database - CINAHL Complete | 22,645 |
| S28 | TI blood glucose | Search modes - Boolean/Phrase | Interface - EBSCOhost Research Databases  Search Screen - Advanced Search  Database - CINAHL Complete | 4,056 |
| S27 | (MH "Blood Glucose") | Search modes - Boolean/Phrase | Interface - EBSCOhost Research Databases  Search Screen - Advanced Search  Database - CINAHL Complete | 41,751 |
| S26 | AB LDL cholesterol | Search modes - Boolean/Phrase | Interface - EBSCOhost Research Databases  Search Screen - Advanced Search  Database - CINAHL Complete | 9,952 |
| S25 | TI LDL cholesterol | Search modes - Boolean/Phrase | Interface - EBSCOhost Research Databases  Search Screen - Advanced Search  Database - CINAHL Complete | 722 |
| S24 | (MH "Lipoproteins, LDL Cholesterol") | Search modes - Boolean/Phrase | Interface - EBSCOhost Research Databases  Search Screen - Advanced Search  Database - CINAHL Complete | 11,191 |
| S23 | AB HDL cholesterol | Search modes - Boolean/Phrase | Interface - EBSCOhost Research Databases  Search Screen - Advanced Search  Database - CINAHL Complete | 9,675 |
| S22 | TI HDL cholesterol | Search modes - Boolean/Phrase | Interface - EBSCOhost Research Databases  Search Screen - Advanced Search  Database - CINAHL Complete | 655 |
| S21 | (MH "Lipoproteins, HDL Cholesterol") | Search modes - Boolean/Phrase | Interface - EBSCOhost Research Databases  Search Screen - Advanced Search  Database - CINAHL Complete | 9,419 |
| S20 | AB Body fat | Search modes - Boolean/Phrase | Interface - EBSCOhost Research Databases  Search Screen - Advanced Search  Database - CINAHL Complete | 13,896 |
| S19 | TI Body fat | Search modes - Boolean/Phrase | Interface - EBSCOhost Research Databases  Search Screen - Advanced Search  Database - CINAHL Complete | 2,336 |
| S18 | AB body weight | Search modes - Boolean/Phrase | Interface - EBSCOhost Research Databases  Search Screen - Advanced Search  Database - CINAHL Complete | 41,190 |
| S17 | TI body weight | Search modes - Boolean/Phrase | Interface - EBSCOhost Research Databases  Search Screen - Advanced Search  Database - CINAHL Complete | 4,966 |
| S16 | (MH "Body Weight") | Search modes - Boolean/Phrase | Interface - EBSCOhost Research Databases  Search Screen - Advanced Search  Database - CINAHL Complete | 34,106 |
| S15 | AB BMI | Search modes - Boolean/Phrase | Interface - EBSCOhost Research Databases  Search Screen - Advanced Search  Database - CINAHL Complete | 60,258 |
| S14 | TI BMI | Search modes - Boolean/Phrase | Interface - EBSCOhost Research Databases  Search Screen - Advanced Search  Database - CINAHL Complete | 3,912 |
| S13 | AB "body mass index" | Search modes - Boolean/Phrase | Interface - EBSCOhost Research Databases  Search Screen - Advanced Search  Database - CINAHL Complete | 72,013 |
| S12 | TI "body mass index" | Search modes - Boolean/Phrase | Interface - EBSCOhost Research Databases  Search Screen - Advanced Search  Database - CINAHL Complete | 8,931 |
| S11 | (MH "Body Mass Index") | Search modes - Boolean/Phrase | Interface - EBSCOhost Research Databases  Search Screen - Advanced Search  Database - CINAHL Complete | 91,768 |
| S10 | AB adiposity | Search modes - Boolean/Phrase | Interface - EBSCOhost Research Databases  Search Screen - Advanced Search  Database - CINAHL Complete | 7,988 |
| S9 | TI adiposity | Search modes - Boolean/Phrase | Interface - EBSCOhost Research Databases  Search Screen - Advanced Search  Database - CINAHL Complete | 3,395 |
| S8 | AB adipose tissue | Search modes - Boolean/Phrase | Interface - EBSCOhost Research Databases  Search Screen - Advanced Search  Database - CINAHL Complete | 10,300 |
| S7 | TI adipose tissue | Search modes - Boolean/Phrase | Interface - EBSCOhost Research Databases  Search Screen - Advanced Search  Database - CINAHL Complete | 4,220 |
| S6 | (MH "Adipose Tissue") | Search modes - Boolean/Phrase | Interface - EBSCOhost Research Databases  Search Screen - Advanced Search  Database - CINAHL Complete | 18,843 |
| S5 | AB fast* N2 (protocol or plan or implement* or regimen or diet or prescription or requirement or lifestyle or intermittent) | Search modes - Boolean/Phrase | Interface - EBSCOhost Research Databases  Search Screen - Advanced Search  Database - CINAHL Complete | 1,410 |
| S4 | TI fast* N2 (protocol or plan or implement* or regimen or diet or prescription or requirement or lifestyle or intermittent) | Search modes - Boolean/Phrase | Interface - EBSCOhost Research Databases  Search Screen - Advanced Search  Database - CINAHL Complete | 505 |
| S3 | AB ((meal* or feed* or eat* or ingestive) N3 (frequency or pattern* or episode* or behavio#r)) | Search modes - Boolean/Phrase | Interface - EBSCOhost Research Databases  Search Screen - Advanced Search  Database - CINAHL Complete | 9,324 |
| S2 | TI (meal* or feed* or eat*or ingestive) N3 (frequency or pattern* or episode* or behavio#r) | Search modes - Boolean/Phrase | Interface - EBSCOhost Research Databases  Search Screen - Advanced Search  Database - CINAHL Complete | 847 |
| S1 | (MH "Eating Behavior") | Search modes - Boolean/Phrase | Interface - EBSCOhost Research Databases  Search Screen - Advanced Search  Database - CINAHL Complete | 19,895 |

**Web of Science:**

((meal or feed* or eat* or ingestive) NEAR/3 (frequency or pattern* or episode* or behavio$r)) or (fast* NEAR/3 (protocol or plan or implement* or regimen or diet or prescription or requirement or lifestyle)) (Topic) and "Body Weight" OR "Body Mass Index" OR "BMI" OR Cholesterol OR "Blood Glucose" OR Glucose OR Diabetes Mellitus or Diabetes OR "Glycated Hemoglobin A" or HbA1c or "HDL cholesterol" or "LDL cholesteroL" or "Adipose Tissue" OR Adiposity or "Body fat" OR Triglycerides (Topic) and (randomised OR randomized OR randomisation OR randomisation OR placebo* OR (random* AND (allocat* OR assign*)) OR (blind* AND (single OR double OR treble OR triple))) (Topic) not (animal or animals or pisces or fish or fishes or catfish or catfishes or sheatfish or silurus or arius or heteropneustes or clarias or gariepinus or fathead minnow or fathead minnows or pimephales or promelas or cichlidae or trout or trouts or char or chars or salvelinus or salmo or oncorhynchus or guppy or guppies or millionfish or poecilia or goldfish or goldfishes or carassius or auratus or mullet or mullets or mugil or curema or shark or sharks or cod or cods or gadus or morhua or carp or carps or cyprinus or carpio or killifish or eel or eels or anguilla or zander or sander or lucioperca or stizostedion or turbot or turbots or psetta or flatfish or flatfishes or plaice or pleuronectes or platessa or tilapia or tilapias or oreochromis or sarotherodon or common sole or dover sole or solea or zebrafish or zebrafishes or danio or rerio or seabass or dicentrarchus or labrax or morone or lamprey or lampreys or petromyzon or pumpkinseed or pumpkinseeds or lepomis or gibbosus or herring or clupea or harengus or amphibia or amphibian or amphibians or anura or salientia or frog or frogs or rana or toad or toads or bufo or xenopus or laevis or bombina or epidalea or calamita or salamander or salamanders or newt or newts or triturus or reptilia or reptile or reptiles or bearded dragon or pogona or vitticeps or iguana or iguanas or lizard or lizards or anguis fragilis or turtle or turtles or snakes or snake or aves or bird or birds or quail or quails or coturnix or bobwhite or colinus or virginianus or poultry or poultries or fowl or fowls or chicken or chickens or gallus or zebra finch or taeniopygia or guttata or canary or canaries or serinus or canaria or parakeet or parakeets or grasskeet or parrot or parrots or psittacine or psittacines or shelduck or tadorna or goose or geese or branta or leucopsis or woodlark or lullula or flycatcher or ficedula or hypoleuca or dove or doves or geopelia or cuneata or duck or ducks or greylag or graylag or anser or harrier or circus pygargus or red knot or great knot or calidris or canutus or godwit or limosa or lapponica or meleagris or gallopavo or jackdaw or corvus or monedula or ruff or philomachus or pugnax or lapwing or peewit or plover or vanellus or swan or cygnus or columbianus or bewickii or gull or chroicocephalus or ridibundus or albifrons or great tit or parus or aythya or fuligula or streptopelia or risoria or spoonbill or platalea or leucorodia or blackbird or turdus or merula or blue tit or cyanistes or pigeon or pigeons or columba or pintail or anas or starling or sturnus or owl or athene noctua or pochard or ferina or cockatiel or nymphicus or hollandicus or skylark or alauda or tern or sterna or teal or crecca or oystercatcher or haematopus or ostralegus or shrew or shrews or sorex or araneus or crocidura or russula or european mole or talpa or chiroptera or bat or bats or eptesicus or serotinus or myotis or dasycneme or daubentonii or pipistrelle or pipistrellus or cat or cats or felis or catus or feline or dog or dogs or canis or canine or canines or otter or otters or lutra or badger or badgers or meles or fitchew or fitch or foumart or foulmart or ferrets or ferret or polecat or polecats or mustela or putorius or weasel or weasels or fox or foxes or vulpes or common seal or phoca or vitulina or grey seal or halichoerus or horse or horses or equus or equine or equidae or donkey or donkeys or mule or mules or pig or pigs or swine or swines or hog or hogs or boar or boars or porcine or piglet or piglets or sus or scrofa or llama or llamas or lama or glama or deer or deers or cervus or elaphus or cow or cows or bos taurus or bos indicus or bovine or bull or bulls or cattle or bison or bisons or sheep or sheeps or ovis aries or ovine or lamb or lambs or mouflon or mouflons or goat or goats or capra or caprine or chamois or rupicapra or leporidae or lagomorpha or lagomorph or rabbit or rabbits or oryctolagus or cuniculus or laprine or hares or lepus or rodentia or rodent or rodents or murinae or mouse or mice or mus or musculus or murine or woodmouse or apodemus or rat or rats or rattus or norvegicus or guinea pig or guinea pigs or cavia or porcellus or hamster or hamsters or mesocricetus or cricetulus or cricetus or gerbil or gerbils or jird or jirds or meriones or unguiculatus or jerboa or jerboas or jaculus or chinchilla or chinchillas or beaver or beavers or castor fiber or castor canadensis or sciuridae or squirrel or squirrels or sciurus or chipmunk or chipmunks or marmot or marmots or marmota or suslik or susliks or spermophilus or cynomys or cottonrat or cottonrats or sigmodon or vole or voles or microtus or myodes or glareolus or primate or primates or prosimian or prosimians or lemur or lemurs or lemuridae or loris or bush baby or bush babies or bushbaby or bushbabies or galago or galagos or anthropoidea or anthropoids or simian or simians or monkey or monkeys or marmoset or marmosets or callithrix or cebuella or tamarin or tamarins or saguinus or leontopithecus or squirrel monkey or squirrel monkeys or saimiri or night monkey or night monkeys or owl monkey or owl monkeys or douroucoulis or aotus or spider monkey or spider monkeys or ateles or baboon or baboons or papio or rhesus monkey or macaque or macaca or mulatta or cynomolgus or fascicularis or green monkey or green monkeys or chlorocebus or vervet or vervets or pygerythrus or hominoidea or ape or apes or hylobatidae or gibbon or gibbons or siamang or siamangs or nomascus or symphalangus or hominidae or orangutan or orangutans or pongo or chimpanzee or chimpanzees or pan troglodytes or bonobo or bonobos or pan paniscus or gorilla or gorillas or troglodytes) (Topic) not Pregnancy or Gestational or Child or Congenital or infant or adolescent or pediatrics or child or (pediatric* or paediatric* or child* or newborn* or congenital* or infan* or baby or babies or neonat* or pre-term or preterm* or premature birth* or NICU or preschool* or pre-school* or kindergarten* or kindergarden* or "elementary school*" or nursery school* or (day care* not adult*) or schoolchild* or toddler* or boy or boys or girl* or middle school* or pubescen* or juvenile* or teen* or youth* or high school* or adolesc* or pre-pubesc* or prepubesc*) (Topic)

**Proquest:**

noft("meal frequency" OR "Eat* frequency") AND noft("Body weight" OR "BMI" OR "Cholesterol" OR "blood glucose") AND noft((randomised OR randomized OR randomisation OR randomisation OR placebo* OR (random* AND (allocat* OR assign*)) OR (blind* AND (single OR double OR treble OR triple)))) NOT ti((animal OR animals OR pisces OR fish OR fishes OR catfish OR catfishes OR sheatfish OR silurus OR arius OR heteropneustes OR clarias OR gariepinus OR fathead minnow OR fathead minnows OR pimephales OR promelas OR cichlidae OR trout OR trouts OR char OR chars OR salvelinus OR salmo OR oncorhynchus OR guppy OR guppies OR millionfish OR poecilia OR goldfish OR goldfishes OR carassius OR auratus OR mullet OR mullets OR mugil OR curema OR shark OR sharks OR cod OR cods OR gadus OR morhua OR carp OR carps OR cyprinus OR carpio OR killifish OR eel OR eels OR anguilla OR zander OR sander OR lucioperca OR stizostedion OR turbot OR turbots OR psetta OR flatfish OR flatfishes OR plaice OR pleuronectes OR platessa OR tilapia OR tilapias OR oreochromis OR sarotherodon OR common sole OR dover sole OR solea OR zebrafish OR zebrafishes OR danio OR rerio OR seabass OR dicentrarchus OR labrax OR morone OR lamprey OR lampreys OR petromyzon OR pumpkinseed OR pumpkinseeds OR lepomis OR gibbosus OR herring OR clupea OR harengus OR amphibia OR amphibian OR amphibians OR anura OR salientia OR frog OR frogs OR rana OR toad OR toads OR bufo OR xenopus OR laevis OR bombina OR epidalea OR calamita OR salamander OR salamanders OR newt OR newts OR triturus OR reptilia OR reptile OR reptiles OR bearded dragon OR pogona OR vitticeps OR iguana OR iguanas OR lizard OR lizards OR anguis fragilis OR turtle OR turtles OR snakes OR snake OR aves OR bird OR birds OR quail OR quails OR coturnix OR bobwhite OR colinus OR virginianus OR poultry OR poultries OR fowl OR fowls OR chicken OR chickens OR gallus OR zebra finch OR taeniopygia OR guttata OR canary OR canaries OR serinus OR canaria OR parakeet OR parakeets OR grasskeet OR parrot OR parrots OR psittacine OR psittacines OR shelduck OR tadorna OR goose OR geese OR branta OR leucopsis OR woodlark OR lullula OR flycatcher OR ficedula OR hypoleuca OR dove OR doves OR geopelia OR cuneata OR duck OR ducks OR greylag OR graylag OR anser OR harrier OR circus pygargus OR red knot OR great knot OR calidris OR canutus OR godwit OR limosa OR lapponica OR meleagris OR gallopavo OR jackdaw OR corvus OR monedula OR ruff OR philomachus OR pugnax OR lapwing OR peewit OR plover OR vanellus OR swan OR cygnus OR columbianus OR bewickii OR gull OR chroicocephalus OR ridibundus OR albifrons OR great tit OR parus OR aythya OR fuligula OR streptopelia OR risoria OR spoonbill OR platalea OR leucorodia OR blackbird OR turdus OR merula OR blue tit OR cyanistes OR pigeon OR pigeons OR columba OR pintail OR anas OR starling OR sturnus OR owl OR athene noctua OR pochard OR ferina OR cockatiel OR nymphicus OR hollandicus OR skylark OR alauda OR tern OR sterna OR teal OR crecca OR oystercatcher OR haematopus OR ostralegus OR shrew OR shrews OR sorex OR araneus OR crocidura OR russula OR european mole OR talpa OR chiroptera OR bat OR bats OR eptesicus OR serotinus OR myotis OR dasycneme OR daubentonii OR pipistrelle OR pipistrellus OR cat OR cats OR felis OR catus OR feline OR dog OR dogs OR canis OR canine OR canines OR otter OR otters OR lutra OR badger OR badgers OR meles OR fitchew OR fitch OR foumart OR foulmart OR ferrets OR ferret OR polecat OR polecats OR mustela OR putorius OR weasel OR weasels OR fox OR foxes OR vulpes OR common seal OR phoca OR vitulina OR grey seal OR halichoerus OR horse OR horses OR equus OR equine OR equidae OR donkey OR donkeys OR mule OR mules OR pig OR pigs OR swine OR swines OR hog OR hogs OR boar OR boars OR porcine OR piglet OR piglets OR sus OR scrofa OR llama OR llamas OR lama OR glama OR deer OR deers OR cervus OR elaphus OR cow OR cows OR bos taurus OR bos indicus OR bovine OR bull OR bulls OR cattle OR bison OR bisons OR sheep OR sheeps OR ovis aries OR ovine OR lamb OR lambs OR mouflon OR mouflons OR goat OR goats OR capra OR caprine OR chamois OR rupicapra OR leporidae OR lagomorpha OR lagomorph OR rabbit OR rabbits OR oryctolagus OR cuniculus OR laprine OR hares OR lepus OR rodentia OR rodent OR rodents OR murinae OR mouse OR mice OR mus OR musculus OR murine OR woodmouse OR apodemus OR rat OR rats OR rattus OR norvegicus OR guinea pig OR guinea pigs OR cavia OR porcellus OR hamster OR hamsters OR mesocricetus OR cricetulus OR cricetus OR gerbil OR gerbils OR jird OR jirds OR meriones OR unguiculatus OR jerboa OR jerboas OR jaculus OR chinchilla OR chinchillas OR beaver OR beavers OR castor fiber OR castor canadensis OR sciuridae OR squirrel OR squirrels OR sciurus OR chipmunk OR chipmunks OR marmot OR marmots OR marmota OR suslik OR susliks OR spermophilus OR cynomys OR cottonrat OR cottonrats OR sigmodon OR vole OR voles OR microtus OR myodes OR glareolus OR primate OR primates OR prosimian OR prosimians OR lemur OR lemurs OR lemuridae OR loris OR bush baby OR bush babies OR bushbaby OR bushbabies OR galago OR galagos OR anthropoidea OR anthropoids OR simian OR simians OR monkey OR monkeys OR marmoset OR marmosets OR callithrix OR cebuella OR tamarin OR tamarins OR saguinus OR leontopithecus OR squirrel monkey OR squirrel monkeys OR saimiri OR night monkey OR night monkeys OR owl monkey OR owl monkeys OR douroucoulis OR aotus OR spider monkey OR spider monkeys OR ateles OR baboon OR baboons OR papio OR rhesus monkey OR macaque OR macaca OR mulatta OR cynomolgus OR fascicularis OR green monkey OR green monkeys OR chlorocebus OR vervet OR vervets OR pygerythrus OR hominoidea OR ape OR apes OR hylobatidae OR gibbon OR gibbons OR siamang OR siamangs OR nomascus OR symphalangus OR hominidae OR orangutan OR orangutans OR pongo OR chimpanzee OR chimpanzees OR pan troglodytes OR bonobo OR bonobos OR pan paniscus OR gorilla OR gorillas OR troglodytes)) NOT ti(Pregnancy OR Gestational OR Child OR Congenital OR infant OR adolescent OR pediatrics OR child OR (pediatric* OR paediatric* OR child* OR newborn* OR congenital* OR infan* OR baby OR babies OR neonat* OR pre-term OR preterm* OR premature birth* OR NICU OR preschool* OR pre-school* OR kindergarten* OR kindergarden* OR "elementary school*" OR nursery school* OR (day care* NOT adult*) OR schoolchild* OR toddler* OR boy OR boys OR girl* OR middle school* OR pubescen* OR juvenile* OR teen* OR youth* OR high school* OR adolesc* OR pre-pubesc* OR prepubesc*))

---

**Clinical Trials.gov**

[**https://clinicaltrials.gov/ct2/results?cond=&term=%22meal+frequency%22+OR+%22eating+frequency%22&cntry=&state=&city=&dist=&Search=Search**](https://clinicaltrials.gov/ct2/results?cond=&term=%22meal+frequency%22+OR+%22eating+frequency%22&cntry=&state=&city=&dist=&Search=Search)

"meal frequency" OR "eating frequency"

**Google Scholar:** [**https://scholar.google.ca/scholar?hl=en&as_sdt=0%2C5&authuser=2&q=%22meal+frequency%22+%22eating+frequency%22+%22Body+Weight%22+%22BMI%22+%22Cholesterol%22+%22Blood+glucose%22+%29+&btnG=**](https://scholar.google.ca/scholar?hl=en&as_sdt=0%2C5&authuser=2&q=%22meal+frequency%22+%22eating+frequency%22+%22Body+Weight%22+%22BMI%22+%22Cholesterol%22+%22Blood+glucose%22+%29+&btnG=)

"meal frequency" "eating frequency" "Body Weight" "BMI" "Cholesterol" "Blood glucose"

**CABI (554) :**

(dD (("meal frequency" OR "eating behaviour" OR "eating patterns" OR "feeding behaviour" OR "feeding habit*" or "snacking" or "gorging" or "fasting" or "ingestive frequency")) AND (("adipose tissue" OR "adiposity" OR "body mass index" OR "BMI" OR "triglycerides" OR "blood glucose" OR "diabetes" OR "body fat" OR "cholesterol" OR "hemoglobin A1C" or "haemoglobin A1C")) AND de:(("adipose tissue" OR "adiposity" OR "body mass index" OR "triglycerides" OR "blood glucose" OR "diabetes" OR "body fat" OR "cholesterol")) AND (("controlled trial" OR randomized OR randomised)) NOT ((pregnan* OR gestation*)) NOT de:((animal* OR mice OR mouse)))e:(("eating behaviour" OR "eating patterns" OR "feeding behaviour" OR "feeding habits")) AN
