# Supplementary material for: The effects of eating frequency on changes in body composition and cardiometabolic health in adults: a systematic review with meta-analysis of randomized trials
Source: Int J Behav Nutr Phys Act. 2023 Nov 14;20:133. doi: 10.1186/s12966-023-01532-z (PMC10647044; doi:10.1186/s12966-023-01532-z)
Supplement: Supplementary file 2 — Additional file 2. Search filters. [file 12966_2023_1532_MOESM2_ESM.docx]

**Supplementary file 2. Search filters**

| FILTER USED | REFERENCE |
| --- | --- |
| (((randomized controlled trial or controlled clinical trial).pt. or (Randomized or placebo or randomly or trial or groups).ab. or (drug therapy).fs.) not (exp animals/ not exp humans/))  <https://docs.google.com/document/d/14E1WhCBvPba1ox4VJwUvUGnAoRcgy-PbUYSS9mEjcs0/edit> | Lefebvre C, Glanville J, Briscoe S, Littlewood A, Marshall C, Metzendorf M-I, Noel-Storr A, Rader T, Shokraneh F, Thomas J, Wieland LS. Technical Supplement to Chapter 4: Searching for and selecting studies. In: Higgins JPT, Thomas J, Chandler J, Cumpston MS, Li T, Page MJ, Welch VA (eds). Cochrane Handbook for Systematic Reviews of Interventions Version 6.1 (updated September 2020). Cochrane, 2020. Available from: www.training.cochrane.org/handbook.  (Cochrane style) |
| (Randomized controlled trial/ or Controlled clinical study/ or random*.ti,ab. or randomization/ or intermethod comparison/ or placebo.ti,ab. or (compare or compared or comparison).ti. or ((evaluated or evaluate or evaluating or assessed or assess) and (compare or compared or comparing or comparison)).ab. or (open adj label).ti,ab. or ((double or single or doubly or singly) adj (blind or blinded or blindly)).ti,ab. or double blind procedure/ or parallel group*1.ti,ab. or (crossover or cross over).ti,ab. or ((assign* or match or matched or allocation) adj5 (alternate or group*1 or intervention*1 or patient*1 or subject*1 or participant*1)).ti,ab. or (assigned or allocated).ti,ab. or (controlled adj7 (study or design or trial)).ti,ab. or (volunteer or volunteers).ti,ab. or human experiment/ or trial.ti.) not (((random* adj sampl* adj7 ("cross section*" or questionnaire*1 or survey* or database*1)).ti,ab. not (comparative study/ or controlled study/ or randomi?ed controlled.ti,ab. or randomly assigned.ti,ab.)) or (Cross-sectional study/ not (randomized controlled trial/ or controlled clinical study/ or controlled study/ or randomi?ed controlled.ti,ab. or control group*1.ti,ab.)) or (((case adj control*) and random*) not randomi?ed controlled).ti,ab. or (Systematic review not (trial or study)).ti. or (nonrandom* not random*).ti,ab. or "Random field*".ti,ab. or (random cluster adj3 sampl*).ti,ab. or ((review.ab. and review.pt.) not trial.ti.) or ("we searched".ab. and (review.ti. or review.pt.)) or "update review".ab. or (databases adj4 searched).ab. or ((rat or rats or mouse or mice or swine or porcine or murine or sheep or lambs or pigs or piglets or rabbit or rabbits or cat or cats or dog or dogs or cattle or bovine or monkey or monkeys or trout or marmoset*1).ti. and animal experiment/) or (Animal experiment/ not (human experiment/ or human/)))  <https://docs.google.com/document/d/1df35fnmVu37svwwVBgnTQvXdCDraO6tAR2Q9Qqx3jes/edit> | Lefebvre C, Glanville J, Briscoe S, Littlewood A, Marshall C, Metzendorf M-I, Noel-Storr A, Rader T, Shokraneh F, Thomas J, Wieland LS. Technical Supplement to [Chapter 4](https://training.cochrane.org/handbook/current/chapter-04): Searching for and selecting studies. In: Higgins JPT, Thomas J, Chandler J, Cumpston MS, Li T, Page MJ, Welch VA (eds). Cochrane Handbook for Systematic Reviews of Interventions Version 6.1 (updated September 2020). Cochrane, 2020. Available from: [www.training.cochrane.org/handbook](http://www.training.cochrane.org/handbook). |
| Exp Child/ or exp infant/ or adolescent/ or exp pediatrics/ or child, abandoned/ or exp child, exceptional/ or child, orphaned/ or child, unwanted/ or minors/ or (pediatric* or paediatric* or child* or newborn* or congenital* or infan* or baby or babies or neonat* or pre-term or preterm* or premature birth* or NICU or preschool* or pre-school* or kindergarten* or kindergarden* or elementary school* or nursery school* or (day care* not adult*) or schoolchild* or toddler* or boy or boys or girl* or middle school* or pubescen* or juvenile* or teen* or youth* or high school* or adolesc* or pre-pubesc* or prepubesc*).mp. or (child* or adolesc* or pediat* or paediat*).jn. | Tjosvold, L., Campbell, SM, Dorgan M.  Filter to Retrieve Pediatric Articles in the OVID Medline Database. John W. Scott Health Sciences Library, University of Alberta. Rev. September 14, 2020  <https://docs.google.com/document/d/1Q3MLfUolWe9q33JdAIzmVK0vi_ieC2Z60e9QvzTgkU8/edit> |
| (exp Animals/ not Humans/) or exp Animal Experimentation/ or exp Models, Animal/ or exp Disease Models, Animal/ or (animal or animals or wildlife or pisces or fish or fishes or catfish or catfishes or sheatfish or silurus or arius or heteropneustes or clarias or gariepinus or fathead minnow or fathead minnows or pimephales or promelas or cichlidae or trout or trouts or char or chars or salvelinus or… | Campbell, Sandy. Filter to Retrieve Studies Related to Animals from the OVID Medline Database. John W. Scott Health Sciences Library, University of Alberta. Rev. October 25, 2022..  <https://docs.google.com/document/d/1mpqZsuCPCbCxmFUf5ndjIUaCCbyu-UmMyWlGGydVgwE/edit> |
| [mh "Child"] or ([mh "Congenital, Hereditary and Neonatal Diseases and Abnormalities"]) or [mh "infant"] or [mh ^"adolescent"] or [mh "pediatrics"] or [mh ^"child, abandoned"] or [mh "child, exceptional"] or [mh ^"child, orphaned"] or [mh ^"child, unwanted"] or [mh ^"minor"] or (pediatric* or paediatric* or child* or newborn* or congenital* or infan* or baby or babies or neonat* or pre-term or preterm* or (premature NEXT birth) or NICU or preschool* or (pre NEXT school*) or kindergarten* or kindergarden* or (elementary NEXT school*) or (nursery NEXT school*) or ((day NEXT care*) not adult*) or schoolchild* or toddler* or boy or boys or girl* or (middle NEXT school*) or pubescen* or juvenile* or teen* or youth* or (high NEXT school*) or adolesc* or (pre NEXT pubesc*) or prepubesc*):ti,ab,kw or (child* or adolesc* or pediat* or paediat*):so | Dennett, Liz. Filter to Retrieve Pediatric Articles in the Wiley Cochrane Library Database. John W. Scott Health Sciences Library, University of Alberta. Rev. February 1, 2020 <https://docs.google.com/document/d/1ZVwv7pvcrehZw1L_nuvLY49ZrF3cIaop-7BwvaJ2zEI/edit> |
