# Supplementary material for: The effects of eating frequency on changes in body composition and cardiometabolic health in adults: a systematic review with meta-analysis of randomized trials
Source: Int J Behav Nutr Phys Act. 2023 Nov 14;20:133. doi: 10.1186/s12966-023-01532-z (PMC10647044; doi:10.1186/s12966-023-01532-z)
Supplement: Supplementary file 3 — Additional file 3. Excluded studies with reasons. [file 12966_2023_1532_MOESM3_ESM.docx]

**Supplementary file 3. Excluded studies with reasons**

| **Year** | **First Author** | **Title** | **Reason(s) for exclusion** |
| --- | --- | --- | --- |
| **1964** | **Fabry** | Frequency of meals - its relation to overweight hypercholesterolaemia + decreased glucose-tolerance | Wrong study design |
| **1966** | **Bortz** | Weight loss and frequency of feeding* | No washout |
| **1968** | **Swindells** | The metabolic response of young women to changes in the frequency of meals | No washout |
| **1970** | **Macdonald** | The influence of frequency of sucrose intake on serum lipid, protein and carbohydrate levels | Wrong intervention - measured sucrose frequency vs meal frequency |
| **1973** | **Debry** | The Effect of Fractionating the Caloric Intake on Weight, Serum Lipids, Post-Prandial Blood Sugar, Serum Insulin and the Oral Glucose Tolerance Test in the Normal Adult | abstract-only |
| **1976** | **Pringle** | Influence of frequency of eating low energy diets on insulin response in women during weight reduction | No washout |
| **1979** | **Van Gent** | Influence of meal frequency on diurnal lipid, glucose and insulin levels in normal subjects on a high fat diet; Comparison with data obtained on a high carbohydrate diet | < 2/52 Intervention |
| **1981** | **Garrow** | The effect of meal frequency and protein concentration on the composition of the weight lost by obese subjects | No washout |
| **1982** | **Dallosso** | Feeding frequency and energy balance in adult males. | No washout |
| **1984** | **Antoine** | Feeding frequency and nitrogen balance in weight-reducing obese women | No washout |
| **1987** | **Wolfram** | Thermogenesis in humans after varying meal time frequency | German-only no translation available |
| **1989** | **Jordan** | Effect of altered feeding patterns on serum lipids and lipoproteins in adult males | No washout (authors not available to comment) |
| **1990** | **Kinabo** | Effect of meal frequency on the thermic effect of food in women | < 2/52 intervention |
| **1991** | **Tai** | Meal size and frequency: effect on the thermic effect of food | < 2/52 intervention |
| **1992** | **Jenkins** | Metabolic advantages of spreading the nutrient load - Effects of increased meal frequency in non-insulin dependent diabetes | < 2/52 intervention |
| **1992** | **Schlundt** | The role of breakfast in the treatment of obesity: a randomised clinical trial | Author unable to provide data for outcomes of interest. |
| **1993** | **Arnold** | Effect of isoenergetic intake of three or nine meals on plasma lipoproteins and glucose metabolism | Author not forthcoming re: washout |
| **1993** | **Verboeket** | Effect of the pattern of food intake on human energy metabolism | < 2/52 intervention |
| **1993** | **Jones** | Meal-frequency effects on plasma hormone concentrations and cholesterol synthesis in humans | < 2/52 intervention |
| **1993** | **Bertelsen** | Effect of meal frequency on blood glucose, insulin, and free fatty acids in NIDDM subjects. | < 2/52 intervention |
| **1994** | **McGrath** | The effects of altered frequency of eating on plasma lipids in free-living healthy males on normal self-selected diets | No washout |
| **1994** | **Arnold** | Metabolic effects of alterations in meal frequency in hypercholesterolaemic individuals. | Author not forthcoming re: washout |
| **1995** | **Verboeket** | Energy expenditure and substrate metabolism in patients with cirrhosis of the liver: effects of the pattern of food intake. | < 2/52 intervention |
| **1995** | **Jenkins** | Effect of Nibbling Versus Gorging on Cardiovascular Risk Factors: Serum Uric Acid and Blood Lipids | < 2/52 intervention / 1-day analysis of previously reported intervention (see Jonkins 1989) |
| **1995** | **Jones** | Meal frequency influences circulating hormone levels but not lipogenesis rates in humans | < 2/52 intervention |
| **1995** | **Segura** | Acute metabolic effects of increased meal frequency in type II diabetes: Three vs six, nine, and twelve meals | < 2/52 intervention |
| **1996** | **Iwao** | Effects of meal frequency on body composition during weight control in boxers. | No - athletic population (boxers) |
| **1997** | **Keim** | Weight loss is greater with consumption of large morning meals and fat-free mass is preserved with large evening meals in women on a controlled weight reduction regimen. | Wrong intervention - No defined meal frequency. No washout |
| **1997** | **Thomsen** | Comparison of the effects of two weeks' intervention with different meal frequencies on glucose metabolism, insulin sensitivity and lipid levels in non-insulin-dependent diabetic patients | No - uneven medication usage between the intervention groups. 1 on metformin and 7 on Sulfonylureas |
| **1997** | **Arnold** | Metabolic effects of alterations in meal frequency in type 2 diabetes. | No washout |
| **1999** | **Speechly** | Acute appetite reduction associated with an increased frequency of eating in obese males. | < 2/52 intervention |
| **2000** | **Johnstone** | Altering the temporal distribution of energy intake with isoenergetically dense foods given as snacks does not affect total daily energy intake in normal-weight men | Wrong intervention - Measured snacks and not E.F. and < 2/52 |
| **2001** | **Taylor** | Compared with nibbling, neither gorging nor a morning fast affect short-term energy balance in obese patients in a chamber calorimeter | < 2/52 intervention |
| **2002** | **Westerterp** | Habitual meal frequency and energy intake regulation in partially temporally isolated men | < 2/52 intervention |
| **2003** | **Volek** | An Isoenergetic Very Low Carbohydrate Diet Improves Serum HDL Cholesterol and Triacylglycerol Concentrations, the Total Cholesterol to HDL Cholesterol Ratio and Postprandial Lipemic Responses Compared with a Low Fat Diet in Normal Weight, Normolipidemic Women | Wrong intervention - studied macronutrient intake not eating frequency |
| **2004** | **De Oliveira** | Meal frequency and serum cholesterol of women in a fruit-or-fiber supplemented diet | Wrong intervention - investigated adding meals (snacks) did not define frequency and no comparator. |
| **2004** | **Lundin** | Effects of meal frequency and high-fibre rye-bread diet on glucose and lipid metabolism and ileal excretion of energy and sterols in ileostomy subjects. | Wrong population - likely to have been affected by surgical intervention |
| **2004** | **Farshchi** | Regular meal frequency creates more appropriate insulin sensitivity and lipid profiles compared with irregular meal frequency in healthy lean women | Wrong intervention - No defined meal frequency in comparator group |
| **2004** | **Farshchi** | Decreased thermic effect of food after an irregular compared with a regular meal pattern in healthy lean women | Wrong intervention - No defined meal frequency in comparator group |
| **2005** | **Farshchi** | Deleterious effects of omitting breakfast on insulin sensitivity and fasting lipid profiles in healthy lean women. | Wrong intervention |
| 2005 | Farshchi | Beneficial metabolic effects of regular meal frequency on dietary thermogenesis, insulin sensitivity, and fasting lipid profiles in healthy obese women | Wrong intervention - No defined meal frequency in comparator group |
| **2005** | **Poston** | weight loss with a meal replacement and meal replacement plus snacks: a randomized trial | Wrong intervention - No defined meal frequency in comparator group |
| **2006** | **Vander Wal** | Effect of a post-dinner snack and partial meal replacement program on weight loss | Wrong intervention - No defined meal frequency |
| **2006** | **Chapelot** | Consequence of omitting or adding a meal in man on body composition, food intake, and metabolism. | < 2/52 intervention |
| **2007** | **Groesz** | An experimental test of the effects of dieting on bulimic symptoms: the impact of eating episode frequency. | Wrong intervention / Wrong outcomes |
| **2008** | **Smeets** | Acute effects on metabolism and appetite profile of one meal difference in the lower range of meal frequency | < 2/52 intervention |
| **2008** | **Solomon** | The effect of feeding frequency on insulin and ghrelin responses in human subjects. | < 2/52 intervention / Wrong outcomes |
| **2010** | **Lara** | An irregular meal pattern for two weeks negatively modifies subjective appetite in overweight subjects | Wrong outcomes (abstract-only) |
| **2010** | **Leidy** | The influence of higher protein intake and greater eating frequency on appetite control in overweight and obese men. | < 2/52 intervention |
| **2010** | **Holmstrup** | Effect of meal frequency on glucose and insulin excursions over the course of a day | < 2/52 intervention |
| **2011** | **Leidy** | The effects of consuming frequent, higher protein meals on appetite and satiety during weight loss in overweight/obese men. | Wrong Outcome - Measured satiety |
| **2012** | **Dougkas** | Differential effects of dairy snacks on appetite, but not overall energy intake | < 2/52 intervention / Wrong outcomes |
| **2012** | **Munsters** | Effects of meal frequency on metabolic profiles and substrate partitioning in lean healthy males | < 2/52 intervention |
| **2013** | **Allirot** | An isocaloric increase of eating episodes in the morning contributes to decrease energy intake at lunch in lean men | < 2/52 intervention |
| **2013** | **Heden** | Meal frequency differentially alters postprandial triacylglycerol and insulin concentrations in obese women. | < 2/52 intervention |
| **2013** | **Ohkawara** | Effects of Increased Meal Frequency on Fat Oxidation and Perceived Hunger | < 2/52 intervention |
| **2014** | **Huseinovic** | Eating frequency, energy intake and body weight... | < 2/52 intervention |
| **2014** | **Piya** | Metabolic endotoxemia: Meal size but not meal frequency matters in metabolically healthy lean and obese subjects | < 2/52 intervention |
| **2014** | **Kanaley** | Alteration of postprandial glucose and insulin concentrations with meal frequency and composition | < 2/52 intervention |
| **2014** | **Kahleova** | The effect of meal frequency on quality of life, beck score of depression, and eating behavior in patients with type 2 diabetes | Wrong outcome(s) |
| **2014** | **Kahleova** | Eating two larger meals a day (breakfast and lunch) is more effective than six smaller meals in a reduced-energy regimen for patients with type 2 diabetes: a randomised crossover study. | No - concerns over medication usage which was inconsistent amongst and between intervention groups. No washout. |
| **2014** | **Koopman** | Hypercaloric Diets With Increased Meal Frequency, but Not Meal Size, Increase Intrahepatic Triglycerides: A Randomized Controlled Trial | Wrong intervention - Undefined meal frequency |
| **2014** | **Salehi** | The effects of 6 Isocaloric Meals Pattern on Blood Lipid Profile, Glucose, Hemoglobin A1c, Insulin and Malondialdehyde in Type 2 Diabetic Patients: A Randomized Clinical Trial | Medication usage affecting outcomes (T2 diabetics) |
| **2015** | **Alhussain** | Influence of the constancy of daily meal pattern on postprandial energy expenditure in healthy weight women | Wrong intervention - Undefined meal frequency in comparator group |
| **2016** | **Alhussain** | Irregular meal-pattern effects on energy expenditure, metabolism and appetite regulation: a randomized controlled trial in healthy normal-weight women | Wrong intervention - Undefined meal frequency in comparator group |
| **2016** | **Mackenzie-Shalders** | Increasing Protein Distribution Has No Effect on Changes in Lean Mass During a Rugby Preseason | Wrong population - athletes AND wrong intervention (did not control for eating frequency only protein intake). |
| **2016** | **Kahleova** | The effect of meal frequency on the fatty acid composition of serum phospholipids in patients with type 2 diabetes. | Secondary study - outcome relevant to out study reported and excluded as per Kahleova 2014. |
| **2016** | **Mok** | Postprandial changes in cardiometabolic disease risk in young Chinese men following isocaloric high or low protein diets, stratified by either high or low meal frequency - a randomized controlled crossover trial | < 2/52 intervention |
| **2016** | **Papakonstantinou** | Effect of meal frequency on glucose and insulin levels in women with polycystic ovary syndrome: a randomised trial | Wrong population - PCOS sufferes have a unique response to insulin associated with condition therefore must exclude. |
| **2016** | **Perrigue** | Higher Eating Frequency Does Not Decrease Appetite in Healthy Adults. | Wrong outcomes - studied appetite-only |
| **2017** | **Nas** | Impact of breakfast skipping compared with dinner skipping on regulation of energy balance and metabolic risk | < 2/52 intervention |
| **2017** | **Belinova** | The effect of meal frequency in a reduced-energy regimen on the gastrointestinal and appetite hormones in patients with type 2 diabetes: A randomised crossover study. | No washout |
| **2017** | **Megson** | Effects of breakfast eating and eating frequency | Wrong study design - Not a controlled trial. Dietary recall only. |
| **2018** | **Kempf** | Individualized Meal Replacement Therapy Improves Clinically Relevant Long-Term Glycemic Control in Poorly Controlled Type 2 Diabetes Patients. | Wrong intervention - meal replacement focus rather than frequency. |
| **2018** | **Meng** | Glycaemic response to three main meals or five smaller meals for patients on rapid-acting insulin. | No - Medication use (rapid acting insulin) |
| **2018** | **Meessen** | Differential Effects of One Meal per Day in the Evening on Metabolic Health and Physical Performance in Lean Individuals | < 2/52 intervention (11-days) |
| **2018** | **Papakonstantinou** | Effects of 6 vs 3 eucaloric meal patterns on glycaemic control and satiety in people with impaired glucose tolerance or overt type 2 diabetes: A randomized trial. | No washout |
| **2018** | **Yao** | Effects of eating frequency on respiratory quotient in patients with liver cirrhosis: a randomized controlled trial | < 2/52 intervention & primarily interested in RQ (wrong outcomes) |
| **2019** | **Jacubowitz** | Reduction in Glycated Hemoglobin and Daily Insulin Dose Alongside Circadian Clock Upregulation in Patients With Type 2 Diabetes Consuming a Three-Meal Diet: A Randomized Clinical Trial. | Medication usage affecting outcomes (insulin dependent diabetics) |
| **2021** | **Taguchi** | Increasing Meal Frequency in Isoenergetic Conditions Does Not Affect Body Composition Change and Appetite During Weight Gain in Japanese Athletes | Competitive rowing athletes eating excess kcals. |
| **2021** | **Alhussain** | Impact of isoenergetic intake of irregular meal patterns on thermogenesis, glucose metabolism, and appetite: a randomized controlled trial. | Wrong intervention - No defined meal frequency in comparator group |
