# Supplementary material for: The effects of eating frequency on changes in body composition and cardiometabolic health in adults: a systematic review with meta-analysis of randomized trials
Source: Int J Behav Nutr Phys Act. 2023 Nov 14;20:133. doi: 10.1186/s12966-023-01532-z (PMC10647044; doi:10.1186/s12966-023-01532-z)

**Supplementary file 4.** Funnel plot analysis of eligible trials assessing the impact of meal frequency on weight change.


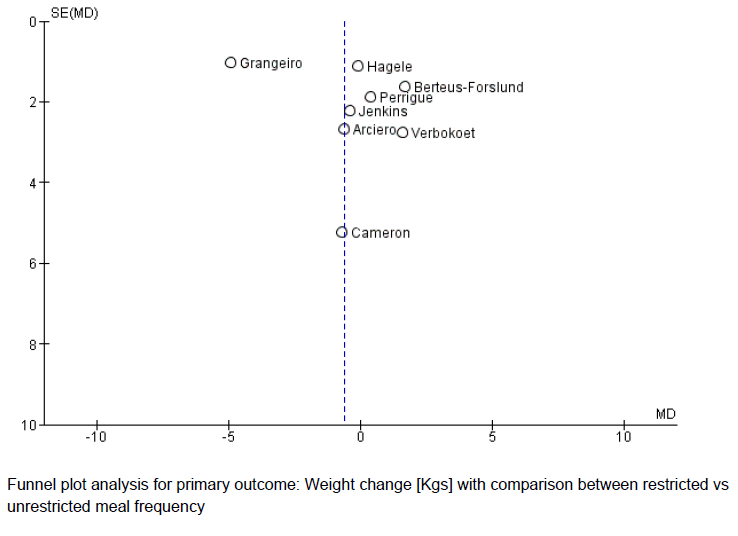

Supplement: Supplementary file 4 — Additional file 4. Funnel plot analysis of eligible trials assessing the impact of meal frequency on weight change. [file 12966_2023_1532_MOESM4_ESM.docx]
