# Supplementary material for: The effects of eating frequency on changes in body composition and cardiometabolic health in adults: a systematic review with meta-analysis of randomized trials
Source: Int J Behav Nutr Phys Act. 2023 Nov 14;20:133. doi: 10.1186/s12966-023-01532-z (PMC10647044; doi:10.1186/s12966-023-01532-z)
Supplement: Supplementary file 5 — Additional file 5. GRADE assessment table with all explanations. [file 12966_2023_1532_MOESM5_ESM.docx]

**Supplementary file 5.** GRADE assessment table with all explanations.

**Question:** Restricted meals (3 or less) compared to unrestricted meals (3 or more) for weight control

| **Certainty assessment** | | | | | | | **№ of patients** | | **Effect** | | **Certainty** | **Importance** |
| --- | --- | --- | --- | --- | --- | --- | --- | --- | --- | --- | --- | --- |
| **№ of studies** | **Study design** | **Risk of bias** | **Inconsistency** | **Indirectness** | **Imprecision** | **Other considerations** | **Restricted meals (3 or less)** | **unrestricted meals (3 or more)** | **Relative (95% CI)** | **Absolute (95% CI)** |  |  |
| **Weight change** | | | | | | | | | | | | |
| 8 | randomised trials | very serious^a^ | serious^b^ | serious^c^ | very serious^d^ | all plausible residual confounding would reduce the demonstrated effect dose response gradient | 138 | 141 | - | MD **0.62 lower** (2.76 lower to 1.52 higher) | ⨁◯◯◯ Very low | CRITICAL |
| **BMI** | | | | | | | | | | | | |
| 5 | randomised trials | very serious^e^ | not serious^f^ | serious^g^ | very serious^h^ | all plausible residual confounding would reduce the demonstrated effect dose response gradient | 113 | 117 | - | MD **0.4 lower** (0.81 lower to 0.02 higher) | ⨁◯◯◯ Very low | IMPORTANT |
| **Fat mass (Kgs)** | | | | | | | | | | | | |
| 5 | randomised trials | very serious^i^ | not serious^j^ | serious^k^ | very serious^l^ | all plausible residual confounding would reduce the demonstrated effect dose response gradient | 72 | 70 | - | SMD **0.28 SD lower** (0.62 lower to 0.05 lower) | ⨁◯◯◯ Very low | IMPORTANT |
| **Tryglyceride** | | | | | | | | | | | | |
| 6 | randomised trials | very serious^m^ | not serious^n^ | serious^o^ | very serious^p^ | all plausible residual confounding would reduce the demonstrated effect dose response gradient | 120 | 123 | - | MD **0.08 lower** (0.08 lower to 0.05 higher) | ⨁◯◯◯ Very low | IMPORTANT |
| **Total cholesterol** | | | | | | | | | | | | |
| 5 | randomised trials | very serious^q^ | serious^r^ | serious^s^ | very serious^t^ | all plausible residual confounding would reduce the demonstrated effect dose response gradient | 94 | 97 | - | MD **0.09 higher** (0.12 lower to 0.29 higher) | ⨁◯◯◯ Very low | IMPORTANT |
| **LDL** | | | | | | | | | | | | |
| 5 | randomised trials | very serious^u^ | not serious^v^ | serious^w^ | very serious^x^ | all plausible residual confounding would reduce the demonstrated effect dose response gradient | 94 | 97 | - | MD **0.05 higher** (0.13 lower to 0.23 higher) | ⨁◯◯◯ Very low | IMPORTANT |
| **HDL** | | | | | | | | | | | | |
| 5 | randomised trials | very serious^y^ | not serious^z^ | serious^aa^ | very serious^ab^ | all plausible residual confounding would reduce the demonstrated effect dose response gradient | 94 | 90 | - | MD **0.05 higher** (0 to 0.09 higher) | ⨁◯◯◯ Very low | IMPORTANT |
| **Glucose** | | | | | | | | | | | | |
| 3 | randomised trials | very serious^ac^ | serious^ad^ | serious^ae^ | very serious^af^ | all plausible residual confounding would reduce the demonstrated effect dose response gradient | 76 | 79 | - | MD **0.09 lower** (0.23 lower to 0.05 higher) | ⨁◯◯◯ Very low | IMPORTANT |
| **Insulin** | | | | | | | | | | | | |
| 3 | randomised trials | very serious^ag^ | not serious^ah^ | serious^ai^ | very serious^aj^ | all plausible residual confounding would reduce the demonstrated effect dose response gradient | 76 | 79 | - | MD **3.38 lower** (6.87 lower to 0.11 higher) | ⨁◯◯◯ Very low | IMPORTANT |

**CI:** confidence interval; **MD:** mean difference; **SMD:** standardised mean difference

#### Explanations

a. All studies had at least one high risk of bias domain, and in most cases this was two domains with high risk of bias. This was enough to suggest there could have been a high risk of effecting the likely outcome, and therefore we have rated down 2-levels.

b. I2 63%, Tau2 = 5.26. Some studies showing non-overlapping confidence intervals with opposing point estimates that have a high variance. Most studies do still hover around the line of no effect but have rated down one level due to lack of consistency.

c. Only 8 studies met our inclusion criteria. Of these six, there was significant differences in the included interventions (e.g. length of intervention, calorie deficit vs neutral, weight status of the patients). The interventions also differed significantly in the variable of interest, namely meal frequency. The populations studied were mostly young to middle aged adults. Thus we have concerns about the applying findings from these studies to all adults.

d. We do not have a defined total amount of weight reduction (in kgs) upon which to base a desired effect size thus making it difficult to calculate the optimal information size. The sample size is small < 400 people total. The CI's for the included trials are wide and most cross the line of no effect. We lack confidence in the precision of these heterogenous trials and therefore rate down two levels.

e. All studies had at least one high risk of bias domain, and in most cases this was two domains with high risk of bias. This was enough to suggest there could have been a high risk of effecting the likely outcome, and therefore we have rated down 2-levels.

f. I2 zero, Tau2 = 0.00. Wide confidence intervals which overlap. Results consistent.

g. The inclusion of studies allowing both weight maintenance (i.e. equicalorific) and weight loss diets diminished our confidence in applying on our findings on the sole benefit of eating frequency specifically.

h. We do not have a defined total amount of reduction in BMI upon which to base a desired effect size thus making it difficult to calculate the optimal information size. The sample size is reasonably small < 400 people total. The CI's for the included trials are wide and most cross the line of no effect. We lack confidence in the precision of these heterogenous trials and therefore rate down two levels.

i. All studies had at least one high risk of bias domain, and in most cases this was two domains with high risk of bias. This was enough to suggest there could have been a high risk of effecting the likely outcome, and therefore we have rated down 2-levels.

j. I2 zero, Tau2 = 0.00. Wide confidence intervals which overlap and minimal effects in either direction, although findings do significantly favor low meal frequency statistically. This is unlikely to be clinically meaningful. Small study number and estimates affected by single study (Hagele et al).

k. The heterogeneity in the calorific intakes at the varying eating frequencies and the split of normal, overweight and obese participants included reduced our confidence in applying the findings to participants intended in our PICO. For Fat mass the measurement tools to assess body also differed considerable between trial, further reducing our confidence in the meta-analysis measure across trials.

l. We do not have a defined total amount of reduction in fat mass (kgs) upon which to base a desired effect size thus making it difficult to calculate the optimal information size. The sample size is small < 400 people total. The CI's for the included trials are wide and most cross the line of no effect. We lack confidence in the precision of these heterogenous trials and therefore rate down two levels.

m. All studies had at least one high risk of bias domain, and in most cases this was two domains with high risk of bias. This was enough to suggest there could have been a high risk of effecting the likely outcome, and therefore we have rated down 2-levels.

n. I2 20%, Tau2 = 0.00. Wide confidence intervals which overlap and minimal effects in either direction.

o. Similar to our concerns around BMI. The heterogeneity in the calorific intakes at the varying eating frequencies and the split of normal, overweight and obese participants included reduced our confidence in applying the findings to participants intended in our PICO.

p. We do not have a defined total amount of reduction triglycerides upon which to base a desired effect size thus making it difficult to calculate the optimal information size. The sample size is small < 400 people total. The CI's for the included trials are wide and ALL cross the line of no effect. We lack confidence in the precision of these heterogenous trials and therefore rate down two levels.

q. All studies had at least one high risk of bias domain, and in most cases this was two domains with high risk of bias. This was enough to suggest there could have been a high risk of effecting the likely outcome, and therefore we have rated down 2-levels.

r. Large variation in point estimates with minimal overlap in confidence intervals. I2 of 63%.

s. The heterogeneity in the calorific intakes at the varying eating frequencies and the split of normal, overweight and obese participants included reduced our confidence in applying the findings to participants intended in our PICO.

t. We do not have a defined total amount of reduction of total cholesterol upon which to base a desired effect size thus making it difficult to calculate the optimal information size. The sample size is small < 400 people total. The CI's for the included trials are wide and most cross the line of no effect. We lack confidence in the precision of these heterogenous trials and therefore rate down two levels.

u. All studies had at least one high risk of bias domain, and in most cases this was two domains with high risk of bias. This was enough to suggest there could have been a high risk of effecting the likely outcome, and therefore we have rated down 2-levels.

v. Moderate variation in point estimates with mostly overlapping confidence intervals. I2 of 59% so high side of moderate. Removal of single study (Jenkins et al) allows for highly consistent results from remaining studies showing no effect in either direction.

w. The heterogeneity in the calorific intakes at the varying eating frequencies and the split of normal, overweight and obese participants included reduced our confidence in applying the findings to participants intended in our PICO.

x. We do not have a defined total amount of reduction (of LDL) upon which to base a desired effect size thus making it difficult to calculate the optimal information size. The sample size is small < 400 people total. The CI's for the included trials are wide and most cross the line of no effect. We lack confidence in the precision of these heterogenous trials and therefore rate down two levels.

y. All studies had at least one high risk of bias domain, and in most cases this was two domains with high risk of bias. This was enough to suggest there could have been a high risk of effecting the likely outcome, and therefore we have rated down 2-levels.

z. Minimal variation in point estimates with overlap in confidence intervals. I2 is very low at 21%.

aa. The heterogeneity in the calorific intakes at the varying eating frequencies and the split of normal, overweight and obese participants included reduced our confidence in applying the findings to participants intended in our PICO.

ab. We do not have a defined total amount of increase in (HDL) upon which to base a desired effect size thus making it difficult to calculate the optimal information size. The sample size is small < 400 people total. The CI's for the included trials are wide and most cross the line of no effect. We lack confidence in the precision of these heterogenous trials and therefore rate down two levels.

ac. All studies had at least one high risk of bias domain, and in most cases this was two domains with high risk of bias. This was enough to suggest there could have been a high risk of effecting the likely outcome, and therefore we have rated down 2-levels.

ad. Small set of studies. Large variation in point estimates between studies. CI's overlap but are extremely wide. Moderate i2 at 42%.

ae. The heterogeneity in the calorific intakes at the varying eating frequencies reduced our confidence in applying the findings to participants intended in our PICO.

af. We do not have a defined total amount of reduction (of glucose) upon which to base a desired effect size thus making it difficult to calculate the optimal information size. The sample size is small < 400 people total. The CI's for the included trials are wide and most cross the line of no effect. We lack confidence in the precision of these heterogenous trials and therefore rate down two levels.

ag. All studies had at least one high risk of bias domain, and in most cases this was two domains with high risk of bias. This was enough to suggest there could have been a high risk of effecting the likely outcome, and therefore we have rated down 2-levels.

ah. Only three studies. One showing a much stronger effect but with wide CI. Other two studies showing very similar effects to each other with large overlap in CIs. I2 is high at 67% but likely due to low number of included studies. Reasoning would indicate this to also be the expected effect from physiological standpoint.

ai. The heterogeneity in the calorific intakes at the varying eating frequencies reduced our confidence in applying the findings to participants intended in our PICO.

aj. We do not have a defined total amount of reduction upon which to base a desired effect size thus making it difficult to calculate the optimal information size. The sample size is very small < 400 people. The CI's for the included trials are wide, and so despite this variable showing a potential significant effect based on our observed data, we lack confidence in the precision of these heterogenous trials and therefore rated down two levels.
