# Supplementary material for: The effects of eating frequency on changes in body composition and cardiometabolic health in adults: a systematic review with meta-analysis of randomized trials
Source: Int J Behav Nutr Phys Act. 2023 Nov 14;20:133. doi: 10.1186/s12966-023-01532-z (PMC10647044; doi:10.1186/s12966-023-01532-z)

**Supplementary file 7**. Sensitivity analysis excluding trials counted drinks-only as meals

Weight change


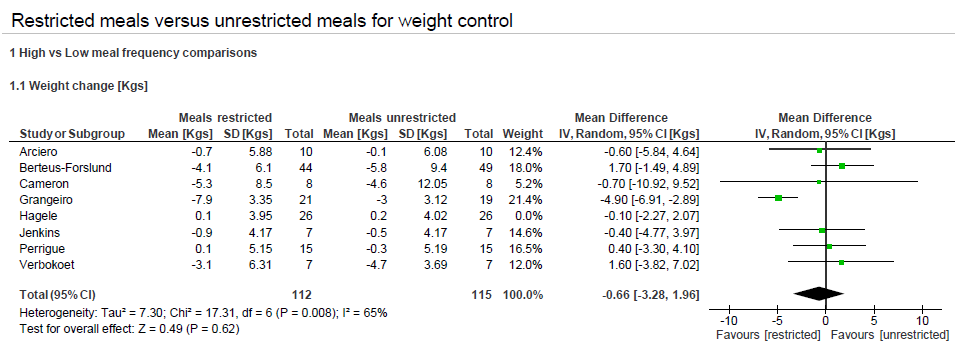


Fat-mass


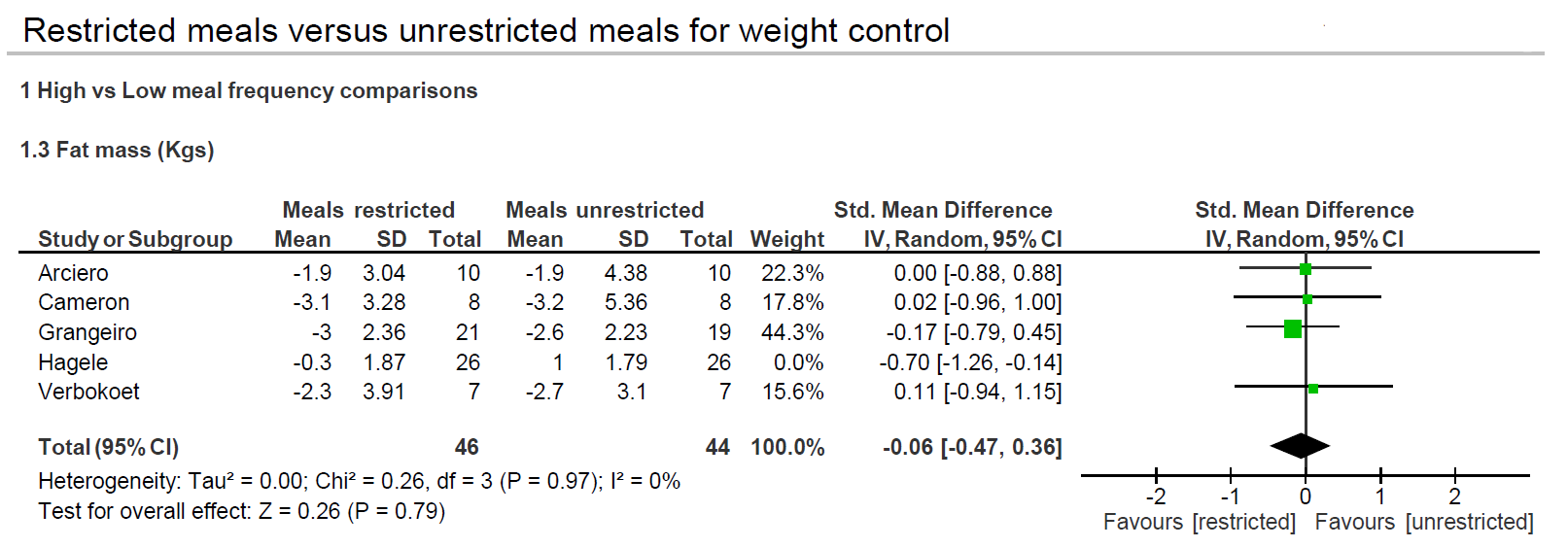


Triglycerides


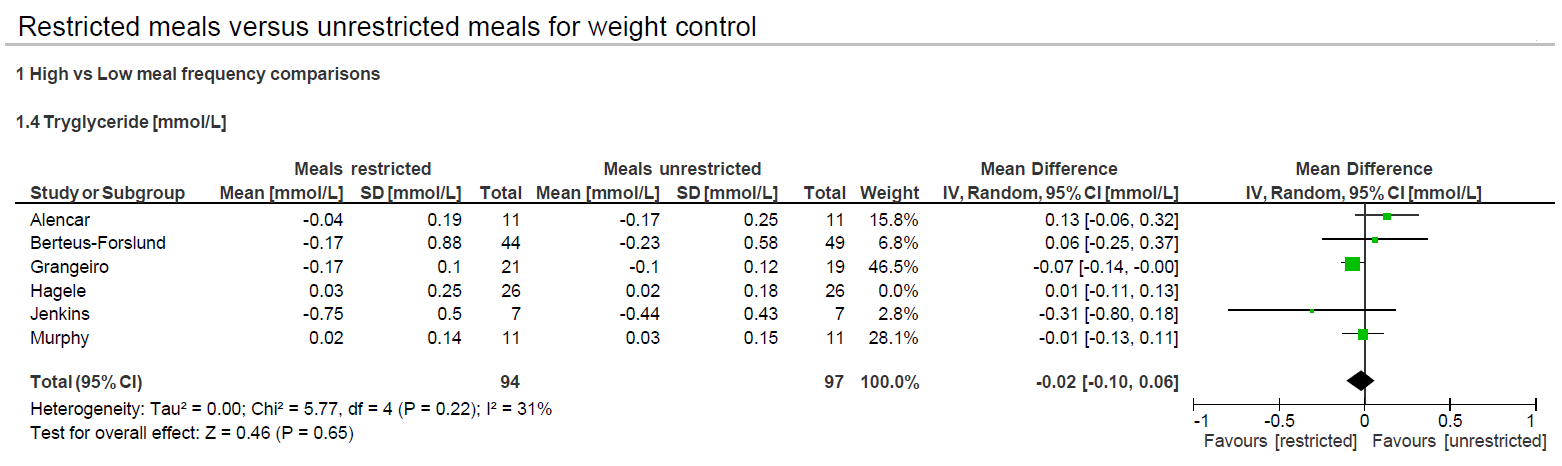

Supplement: Supplementary file 7 — Additional file 7. Sensitivity analysis excluding trials counted drinks-only as meals [file 12966_2023_1532_MOESM7_ESM.docx]
